# Comparative analysis of virus-host interactions caused by a virulent and an attenuated duck hepatitis A virus genotype 1

**DOI:** 10.1371/journal.pone.0178993

**Published:** 2017-06-14

**Authors:** Xumin Ou, Sai Mao, Jingyu Cao, Anchun Cheng, Mingshu Wang, Dekang Zhu, Shun Chen, Renyong Jia, Mafeng Liu, Kunfeng Sun, Qiao Yang, Ying Wu, Xiaoyue Chen

**Affiliations:** 1 Institute of Preventive Veterinary Medicine, Sichuan Agricultural University, Wenjiang, Chengdu City, Sichuan, People′s Republic of China; 2 Key Laboratory of Animal Disease and Human Health of Sichuan Province, Sichuan Agricultural University, Wenjiang, Chengdu City, Sichuan, People′s Republic of China; 3 Avian Disease Research Center, College of Veterinary Medicine, Sichuan Agricultural University, Wenjiang, Chengdu City, Sichuan, People′s Republic of China; The University of Melbourne, AUSTRALIA

## Abstract

Because of their better immunogenicity and the improved protection they afford, live attenuated vaccines derived from serial passaging in an abnormal host are widely used to protect humans or animals from certain pathogens. Here, we used a virulent and a chicken embryo-attenuated duck hepatitis A virus genotype 1 to compare the different regulated immune responses induced by viruses with differing virulence. In this study, the attenuated strains had lower protein expression levels than the virulent strains as identified by immunohistochemistry. This may be caused by apparent codon usage bias selected during passage. Furthermore, lower translation efficiency led to decreased viral replication, which is highly dependent on non-structural viral protein expression. Although the two strains had differing levels of virulence, both could induce strong innate immune responses and robust Tc or Th cell populations during the early stages of the immune response. However, due to fixed single nucleotide polymorphisms (SNPs) selected by passage, the virulent and attenuated strains may induce differing immune responses, with stronger Tc cell immunity induced by the attenuated strain in the spleen and thymus and stronger Tc cell immunity induced by the virulent strain in the liver, lung, bursa of Fabricius and Harderian gland. Four immune related genes (RIG-1, MDA5, IFN-β, and IL-6) were highly differentially expressed in the Harderian gland, bursa of Fabricius and thymus. This study has provided further information about differences in virus-host interactions between duck hepatitis A viruses of differing virulence.

## Introduction

Many genetic diseases and malignant tumors in humans are caused by genetic variations, such as sickle cell anemia [1], hemophilia [2], breast cancer [3], small-cell lung cancer [4], etc. Genetic variations are also found in prokaryotes and viruses under selection pressure by medicines, vaccines or an unsuitable microenvironment, which leads to strain diversity or genotypes with different levels of virulence. However, diverse immune regulation induced by virulence in viruses is not entirely understood, at least in ducks. Specifically, viral virulence can be attenuated by serial passaging in an unsuitable host, and this theory was tested approximately a half century ago with vaccines, such as the Oral polio vaccine (OPV), attenuated dengue vaccine, Measles vaccine and Mumps vaccine [5–8]. In this study, virulent and chicken embryo-attenuated strains of Duck Hepatitis A Virus genotype 1 were used to compare the diverse immune regulation caused by the diversity of virulence.

Duck viral hepatitis (DVH) is a highly fatal, rapidly infectious disease in ducklings characterized by swollen livers mottled with hemorrhages [9]. Duck hepatitis virus (DHV) was first reported in Long Island, New York in the United States by Levine and Hofstad (1945) and was later transmitted to England, Germany, Canada, Japan and China [10]. The disease can cause mortality rates up to 95% in young ducklings within 1 week but is not fatal in mature ducks [9]. Three genotypes of DHV type I (renamed Duck Hepatitis A Virus, DHAV) and two astroviruses (DHV types II and III) cause this disease. DHAV is classified in the new genus *Avihepatovirus* of the family *Picornaviridae* [9, 11], and DHV types II and III are known as Duck Astroviruses. No antigenic relationships have been found among DHV types I, II and III based on serum neutralization testing [9]. However, based on their genetic sequences, DHAVs are grouped into the following three genotypes: DHAV-1 (classical genotype 1) [12, 13], DHAV-2 (isolate from Taiwan) [14] and DHAV-3 (new isolates from South Korea and China) [15]. Previously, DHAV-2 and DHAV-3 have been shown to be genetically and serologically distinct from DHAV-1, but recent studies indicated that DHAV-1 and DHAV-3 had limited cross-neutralization [9, 16–18]. To our knowledge, the higher mortality in ducklings following DHAV-1 infection is currently attributed to their incompletely developed immune systems [19]. This conclusion was formed from observations of more severe pathological changes (hemorrhage and edema) in the duck embryo than in ducklings and the apparently enlarged livers mottled with hemorrhages that can be observed in ducklings but not in mature ducks [9]. Due to these advantages, mature ducks have been suggested as useful models to evaluate vaccines and investigate avian immune responses.

Birds evolved from a common reptilian ancestor and have inherited many immunological mechanisms in common with mammals, but they also have developed a number of quite distinct immune organs, such as the bursa of Fabricius, thymus and Harderian gland. Generally, innate immunity, the first line of host defense against pathogens, is mainly triggered by macrophages and dendritic cells (DCs), whereas acquired immunity is characterized by specificity and memory generated by a vast repertoire of lymphocytes bearing antigen-specific receptors [20]. Notably, activation of the innate immune response can be a prerequisite for the triggering of acquired immunity [21–23]. Macrophages, DCs and Natural killer cells harbor a battery of Pattern Recognition Receptors (PRRs) responsible for the recognition of pathogen-associated molecular patterns (PAMPs) and subsequently mediate signaling cascades to induce the expression of a variety of overlapping and unique genes involved in inflammatory and immune responses [21]. For example, both TLR3 and TLR7, located in endosomes, are exposed to dsRNA and ssRNA during the viral uncoating or replication processes, respectively [24]. RIG-I and MDA5 detect RNA ligands derived from short dsRNA and long dsRNA and induce the IFN response [25, 26]. Once the virus is recognized by those sensors, pro-inflammatory cytokines and interferons (IFNs) are secreted by innate immune cells [27, 28]. Thereafter, MHC-I or MHC-II molecules timely expressed on the surface of antigen presenting cells (APCs) are responsible for activating CD8+ cytotoxic (Tc) or CD4+ T helper (Th) cells, respectively [29, 30]. Subsequently, DCs and naïve lymphocytes chemotactically migrate to secondary lymphoid tissues following exposure to chemokines [31]. BAFF/BLyS, a member of the tumor necrosis factor (TNF) family of proteins, is essential for B cell survival and plays an important role in regulating class switch recombination (CSR) of immunoglobulins [32].

Previously, Asplin and Reuss reported a loss of DHAV-1 pathogenicity in ducklings after passaging the virus in chicken embryos [33, 34]. The aim of this study was to characterize the diverse immune responses regulated by a virulent strain and an attenuated strain of DHAV-1 and to identify how virulence affects the immunological effects of DHAV-1 in mature ducks. To address those questions, the immunological effects of the virulent DHAV-1 H strain and the attenuated DHAV-1 CH60 strain were compared in this study 2 days after inoculation. In addition, the fixed SNPs in chicken embryo attenuated strains that resulted from serial passaging in chicken embryos were also identified.

## Materials and methods

### Ethics statement

This study was performed in strict accordance with the recommendations of the ARRIVE guidelines (http://www.nc3rs.org.uk/arrive-guidelines). The animal experiments were approved by the committee of experiment operational guidelines and animal welfare of Sichuan Agriculture University, China (approval permit number XF2014-18). All ducks were handled in compliance with the animal welfare regulations and maintained according to standard protocols. The animals were anesthetized by intravenous injection of sodium pentobarbital (40 mg/kg). Then, the ducks were quickly sacrificed and blood and tissues harvested. There were no surgeries.

### Viruses

The DHAV-1 CH60 attenuated vaccine (GenBank: KU923754.1) and a DHAV-1 H strain (GenBank: JQ301467.1) were selected to investigate their differential effects on immune responses. The attenuated strain, which was derived from the DHAV-1 CH strain in the allantoic cavities of 9-day-old specific pathogen free (SPF) chicken embryos after 60 passages, is a commercial vaccine developed by our laboratory. We named it the DHAV-1 CH strain when it was propagated in 1-day-old susceptible ducklings. This strain was renamed the DHAV-1 H strain when it was subsequently propagated for one to three generations in 9- to 11-day-old duck embryos. The embryos that died within 24 h post-infection (hpi) were discarded, and those that died at 36–72 hpi were harvested. The homogenates of chicken embryonic bodies and the allantoic fluids from the duck embryos were stored at -80°C until use. The CH60 strain titer was determined to be 10^−6.55^ chicken embryo lethal median dose (ELD_50_)/0.2 ml. Virus at a concentration of 4.56 × 10^8^ copies/ml, as determined by quantitative real-time PCR (qPCR), was used to infect ducks [35]. To identify the differences caused by serial passaging, comparative analysis of the other representative strains from virulent and chicken embryo attenuated strains were selected to address the potential reasons driving genetic variations (S1 Table).

### Codon usage indices and multiple sequence alignment

The Relative Synonymous Codon Usage (RSCU) was calculated using CodonW (http://www.molbiol.ox.ac.uk/cu, version 1.4.2) with *Saccharomyces cerevisiae* as a reference. The RSCUs used by the virulent and attenuated strains were visualized using hemi with hierarchical clustering analysis (http://hemi.biocuckoo.org/) (S3 Table). The conforming nucleotide substitutions were identified by Mega 6.0 using Cluster W method [36].

### Animals

Fifteen 160-day-old female Peking ducks (*Anas platyrhynchos domesticus*) were purchased from commercial hatcheries at Mianyang city in Sichuan province and housed in isolation under the Laboratory Animal Management Regulations of Sichuan Agriculture University. The ducks were obtained free of virus-specific antibodies determined by an indirect Elisa assay before exposure to DHAV (vaccine strain or virulent strain) [37]. The ducks were provided access to feed and water *ad libitum* before and after virus injection. The behavior and diet were monitored every 12 hours (8:00 am and 20:00 pm). There were no accidental deaths during this study.

### Experimental design

Fifteen ducks were randomly divided into three groups (n = 5). Group 1 (CH60 strain) and group 2 (H strain) received 1 ml of the virus by intramuscular injection, whereas group 3 was injected with an equal volume of 0.85% physiological saline as a negative control. The two viral strains at 4.56 ×10^8^ copies/ml, as determined by qPCR, were used to infect ducks. Those ducks were humanely sacrificed 2 days after inoculation. One hundred milligrams of each specimen was weighed, rinsed in cold sterile saline and immediately cryopreserved in liquid nitrogen until processed for RNA isolation.

### RNA isolation and cDNA preparation

Total RNA was isolated from 100 mg of each organ (n = 5) and 150 μl of blood (n = 5) using the RNAiso Reagent (TaKaRa, Japan) according to the manufacturer’s protocols. Briefly, 100 mg of each tissue was processed into a fine powder in a ceramic mortar with a sufficient amount of liquid nitrogen followed by addition of 1000 μl of RNAiso Reagent and extraction with 200 μL of chloroform with intense shaking for 15 s. The nucleic acids were suspended by centrifugation (12,000 × *g* at 4°C for 15 min), and then 500 μL of isopropanol was added. The RNA was then pelleted by centrifugation (12,000 × *g* at 4°C for 15 min) and washed with 75% ethanol. After air drying, the RNA pellet was resuspended in 50 μL of diethyl pyrocarbonate (DEPC)-treated water and stored at −70°C until use. The total RNA was detected spectrophotometrically using the Smartspec-3000 (Bio-Rad, USA) and agarose gel electrophoresis to confirm the quality.

The RNA isolated from each specimen was needed to detect immune-related gene expression and was treated with 1.0 μL of gDNA Eraser (TaKaRa, Japan) for two min at 42°C to remove the potential contaminating genomic DNA (gDNA) and then used to carry out a reverse transcription to produce cDNA with the PrimeScript^TM^ RT Reagent Kit according to the manufacturer’s instructions (TaKaRa, Japan).

### qPCR

The expression of seventeen immune-related genes (IL-1β, IL-2, IL-4, IL-6, IFN-α, IFN-β, IFN-γ, MHC-I, MHC-II, CCL19, CCL21, BAFF, TLR3, TLR7, β-defensin, RIG-1 and MDA5) and a housekeeping gene glyceraldehyde-3-phosphate dehydrogenase (GAPDH) were detected by qPCR in the most frequently targeted tissues, such as blood, liver, spleen, lung, kidney, Harderian gland, bursa of Fabricius and thymus. The primer sequences used were previously published [38, 39]. All primers used in this study are shown in S2 Table. The expression levels of mRNA transcripts were determined by qPCR using the SYBR^®^Premix Ex Taq™ II (Tli RNaseH Plus) Kit (Takara). To evaluate the error range, the Ct values of liver samples were detected three times using the following protocol. The amplification procedure was performed in a 20-μL reaction volume containing 8 μM of each primer and 2 μl of cDNA. The following thermal cycling conditions were used: PCR initial activation at 95°C for 30 s, followed by 45 cycles of denaturation at 95°C for 5 s and annealing and extension at 58.2°C for 30 s.

### HE staining and immunohistochemistry

The tissues from the Harderian gland, bursa of Fabricius, thymus, liver, spleen, lung and kidney from the same samples used for transcriptional analysis were fixed in 4% paraformaldehyde, dehydrated, embedded in paraffin, sectioned into 4-μm thick sections and stained with hematoxylin and eosin (HE) using standard procedures [39]. Those sections were also used for standard IHC staining. Briefly, paraffin-embedded tissues were deparaffinized in xylene and rehydrated in graded alcohols. For antigen retrieval, slides were boiled in Tris/EDTA pH 9.0 for 20 min. A solution of 0.01 M HCl was used to block endogenous alkaline phosphatase for 15 min at room temperature. The slides were incubated in 5% BSA blocking solution followed by overnight incubation with rabbit anti-DHAV polyclonal antibody (1:40 dilution), rabbit anti-2A2 polyclonal antibody (1:20 dilution), rabbit anti-2A3 polyclonal antibody (1:20 dilution), rabbit anti-3C polyclonal antibody (1:20 dilution) and rabbit anti-3D polyclonal antibody (1:20 dilution). Then, the slides were incubated with alkaline phosphatase-conjugated goat anti-rabbit IgG (1:1000 dilution, Life Technology) for 30 min at 37°C. The positive stains were visualized with BCIP/NBT solution for 20 min at room temperature and counterstained with nuclear fast red. Additionally, DHAV and CD4+ or CD8+ positive T cells were double stained by first incubating with a cocktail of rabbit anti-DHAV polyclonal antibody and mouse anti-duck CD4 or CD8a monoclonal antibody (1:40 dilution and 1:200 or 1:100 dilution, respectively) followed by a mixture of alkaline phosphatase and HRP coupled goat anti-rabbit and donkey anti-mouse secondary antibodies. Then, the positive cells were visualized with DAB solution for 10 min and permanent Red solution for 15 min at room temperature and counterstained with hematoxylin.

### Statistical analyses

Relative gene expression data were analyzed using the 2^-ΔΔCt^ method by comparison with the control group injected with 1 ml normal saline (NS) [40], and the ΔCt values were determined by subtracting the average Ct values of the endogenous control gene GAPDH from the average Ct values of the target genes. The histogram was generated using GraphPad Prism 5 software. Statistically significant differences among groups were determined by one-way ANOVA using SPSS 20.0 statistical software, and *post hoc* analysis (Fisher's least significant difference, LSD) was utilized to analyze the differences among the means of transcripts grouped by CH60 and H strains (*P* < 0.05).

## Results

### Apparent codon usage bias was caused by serial passaging in chicken embryos

**Fig 1:**
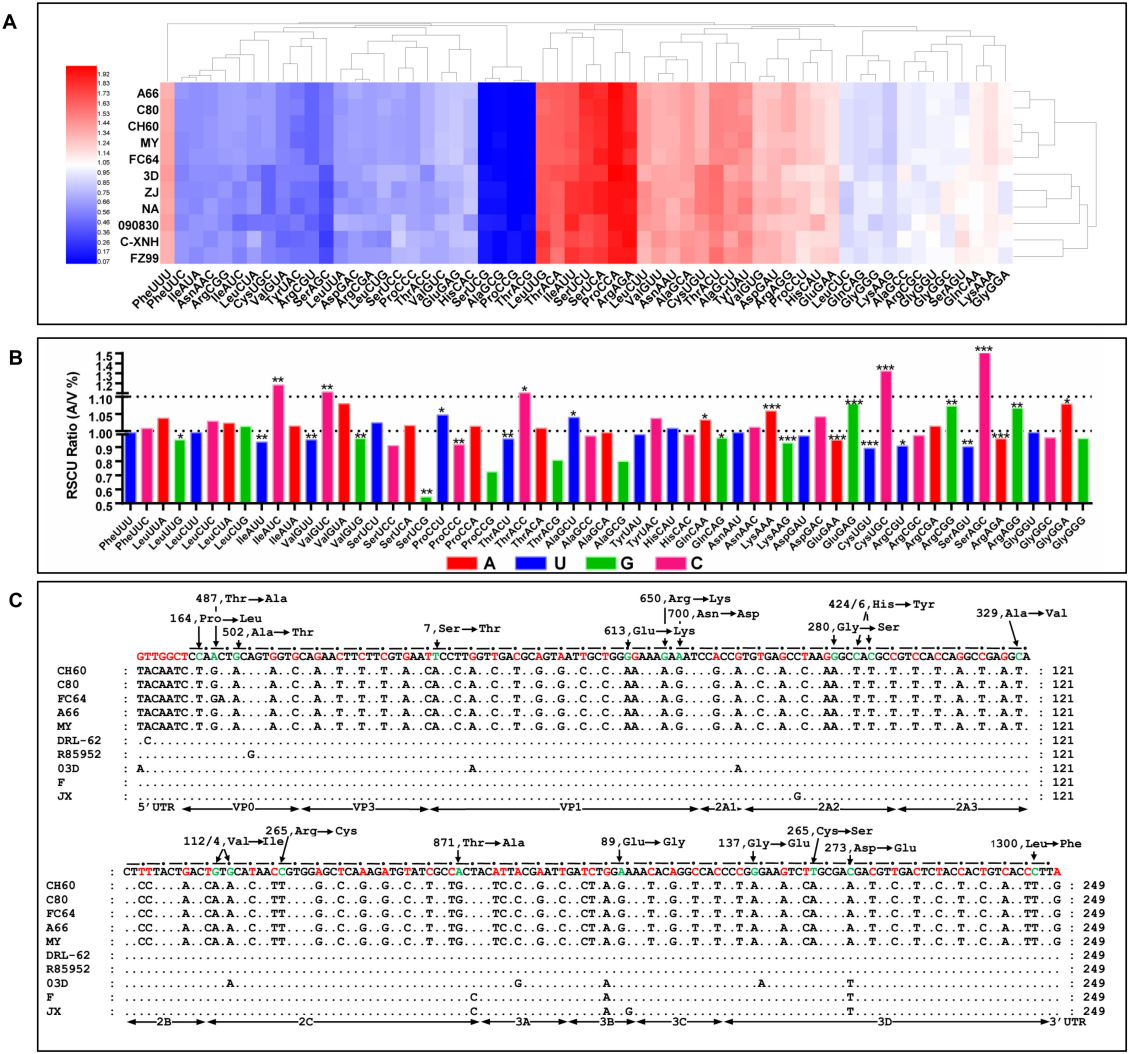
Codon usage bias analysis and fixed SNPs in attenuated and virulent strains. A) RSCUs used in virulent and chick embryo attenuated strains were subject to hierarchical cluster analysis according to their Euclidean distances (Hemi software). The results indicated that the RSCU was apparently changed in the process of passage in chick embryos. B) The significance of differences of RSCU between the chick embryo attenuated strains and the virulent strains were analyzed by Student-t test. The ratios (A/V) at 1.0 and 1.1 are displayed as two dash lines. *P < 0.05, **P < 0.01 or ***P < 0.001 indicate the level of statistical significance of the differences between different groups. C) Fixed SNPs in the genomes of virulent strains and chick embryo attenuated strains. The conserved nucleotides used by virulent strains are listed on the first line, which corresponds to the dark spots. Red color indicated synonymous mutations or mutations in UTR. Green color indicated missense mutations, which are indicated at each site.

The patterns of codon usage in the serial chick embryo-attenuated viral strains were apparently changed in the hierarchical cluster results (Fig 1A). To further elucidate the significance levels of each codon, a comparative analysis indicated that almost half (27/59, 45.76%) of the codon usage bias was significantly changed (Fig 1B). The synonymous and missense mutations and the fixed SNPs in the UTR from the chick embryo passaged strains and virulent strains are shown in Fig 1C. The number of SNPs in the 3^rd^ codon position was 61, including 14A, 8G, 20T and 19C. These data indicate that the patterns of codon usage in serial chick embryo attenuated strains were significantly different than those of the virulent strains.

### TLR7 and alternatively activated TLR3, RIG-1 and MDA5 are involved in early PRR recognition

**Fig 2:**
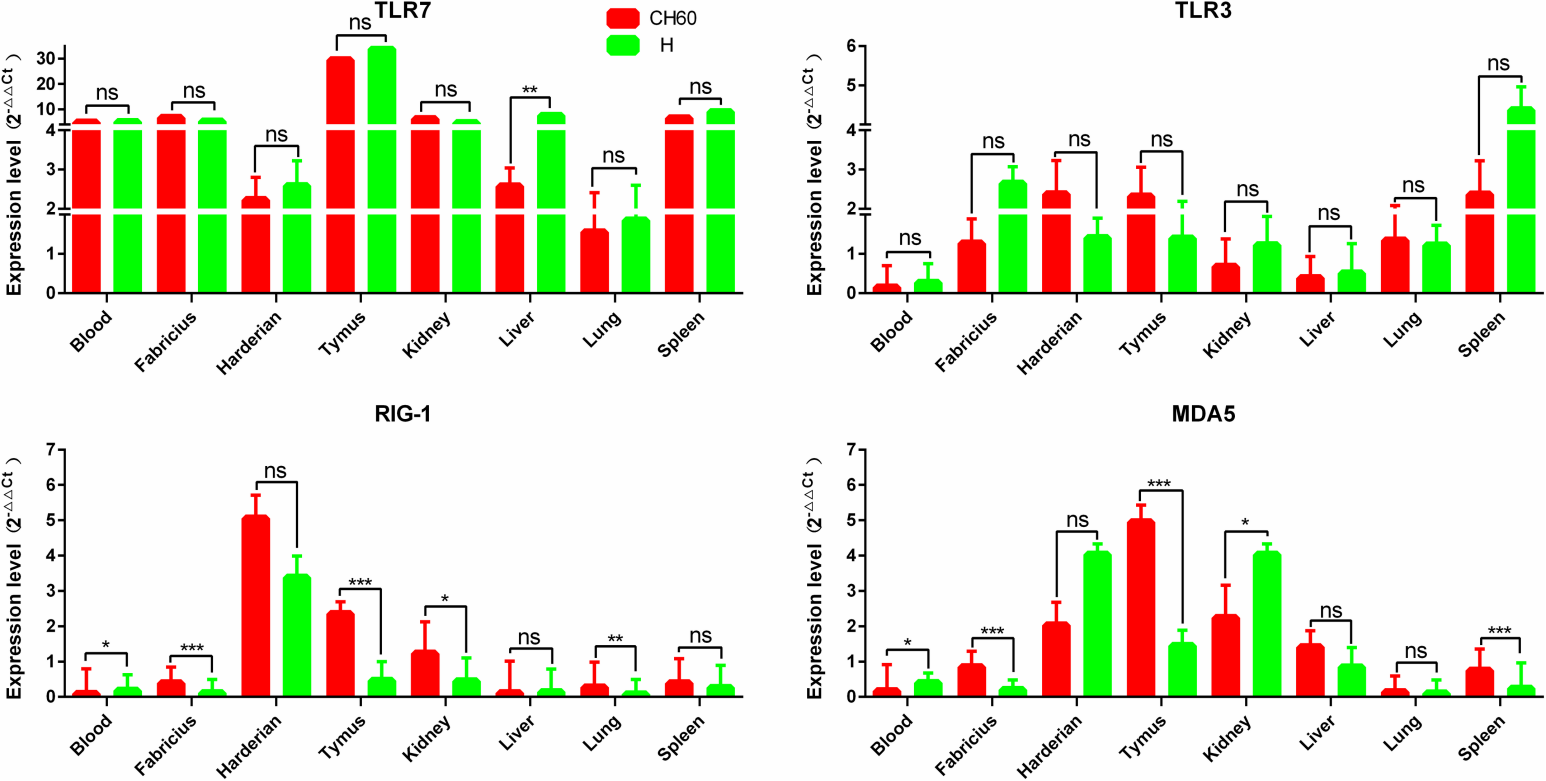
Expression pattern of PRRs in different organs. The relative expression levels of TLR7, TLR3, RIG-1 and MDA5 were calculated by the 2^-ΔΔCt^ method. The error bars represent standard errors of the mean. Statistically significant differences between the mean gene expression levels in different organs were determined by one-way ANOVA (*P* < 0.05). *P < 0.05, **P < 0.01 or ***P < 0.001 indicate the level of statistical significance of differences between different groups.

Due to the crucial role that PRRs play in innate immunity, TLR7, TLR3, RIG-1 and MDA5 expression levels in different tissues were compared between the CH60 and H strains. TLR7 expression was highly up-regulated in all selected tissues except in the lung. By comparison, the expression levels of TLR3, RIG-1 and MDA5 were selectively up-regulated, especially in immune organs such as the Harderian gland, thymus, bursa of Fabricius and spleen (Fig 2). Specifically, the expression of TLR3, RIG-1 or MDA5 in the spleen, Harderian gland and kidney were consistently up-regulated in both the CH60 and H strains in the infected tissues. Moreover, RIG-1 and MDA5 were highly expressed in the CH60 strain infected thymus but not in the thymus infected with H strains (*P*<0.01).

### Interferons and interleukins are highly activated by DHAV-1 CH60 and H strains

**Fig 3:**
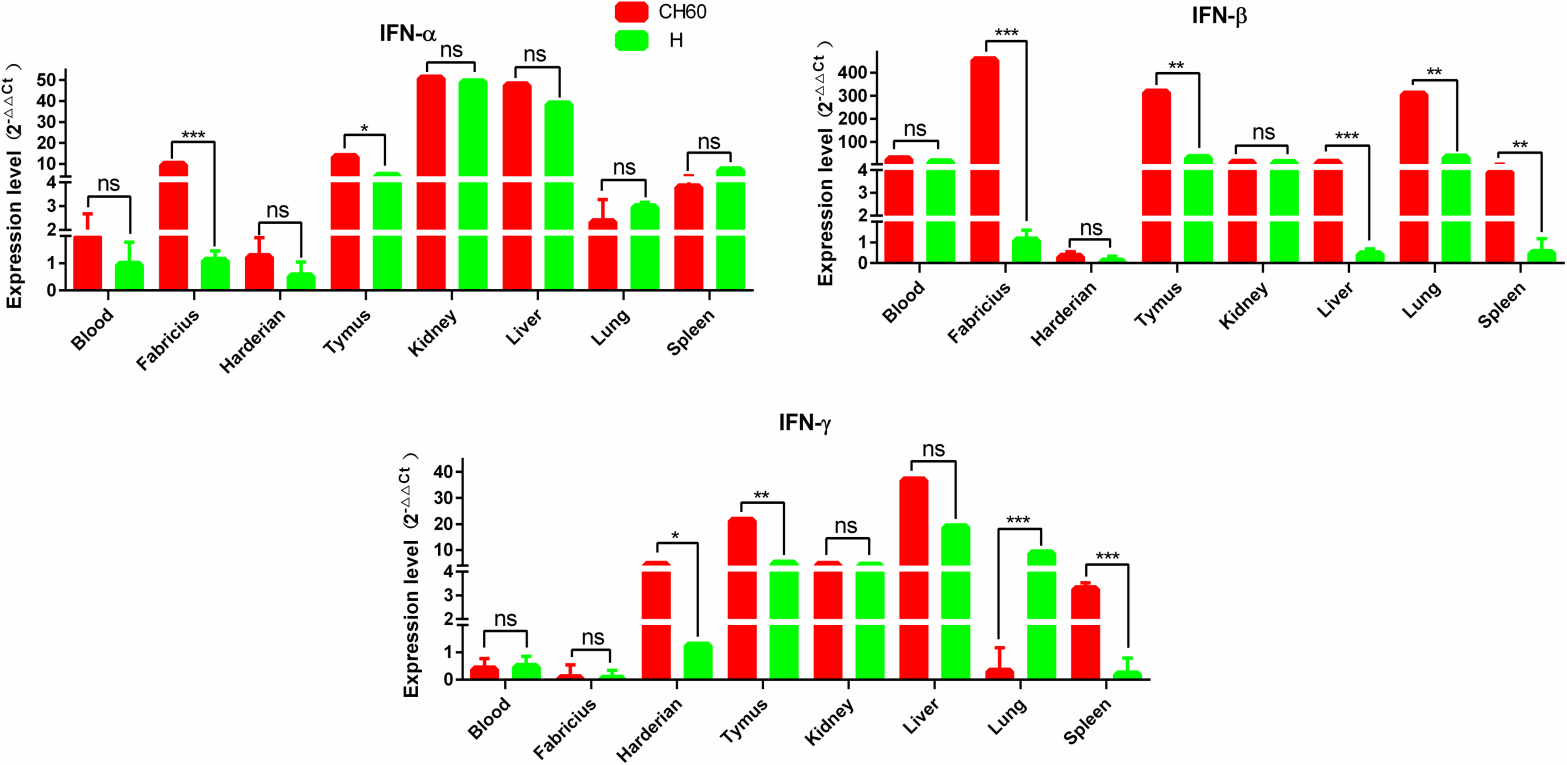
Expression patterns of interferons in different organs. The relative expression level of IFN-α, IFN-β and IFN-γ were calculated using the 2^-ΔΔCt^ method. The error bars represent standard errors of the mean. Statistically significant differences between the mean gene expression levels in different organs were determined by one-way ANOVA (*P* < 0.05). *P < 0.05, **P < 0.01 or ***P < 0.001 indicate the level of statistical significance of differences between different groups.

**Fig 4:**
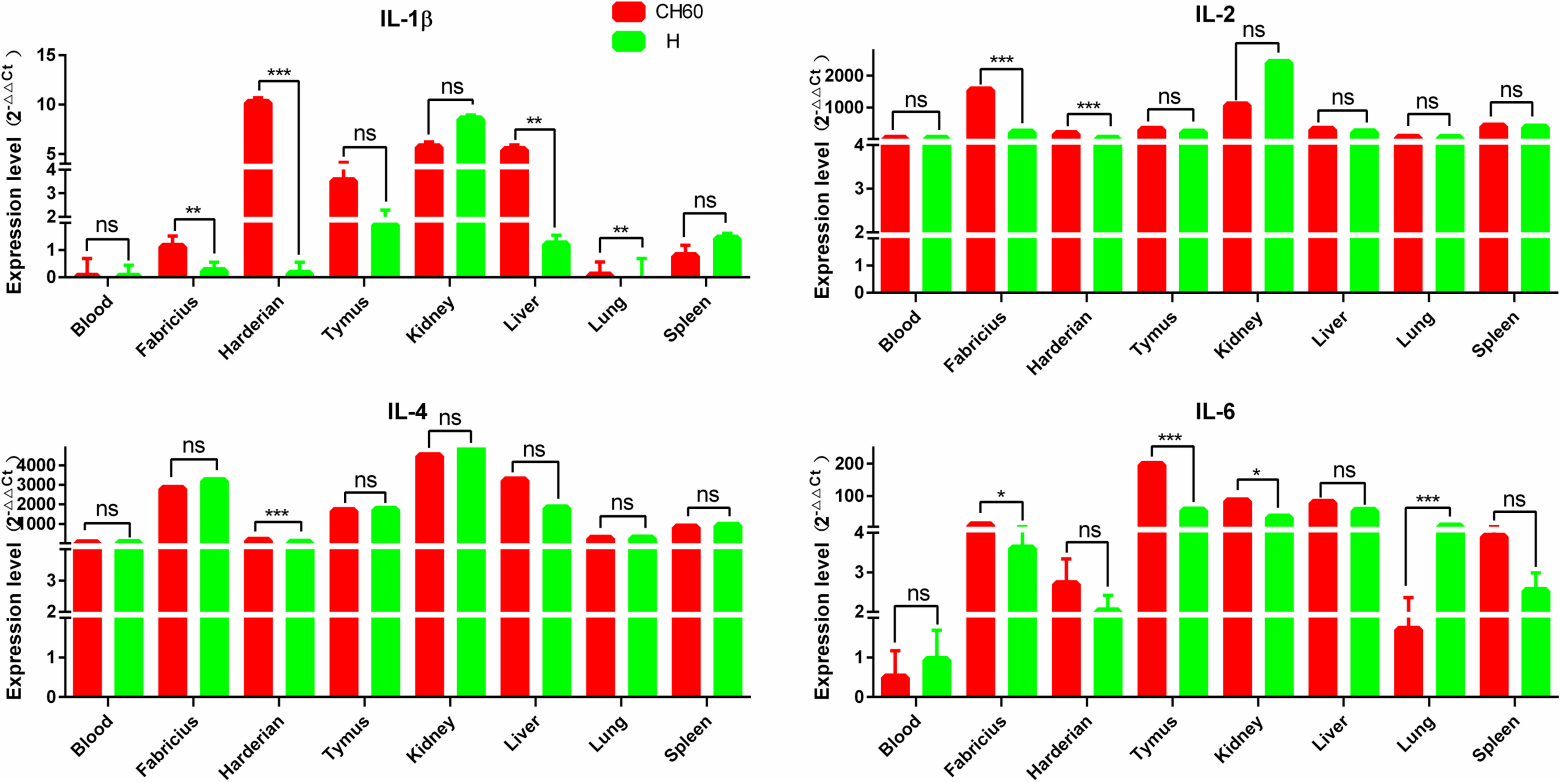
Expression patterns of interleukins in different organs. The relative expression levels of IL-1β, IL-2, IL-4 and IL-6 were calculated using the 2^-ΔΔCt^ method. The error bars represent standard errors of the mean. Statistically significant differences between the mean gene expression levels in different organs were determined by one-way ANOVA (*P* < 0.05). *P < 0.05, **P < 0.01 or ***P < 0.001 indicate the level of statistical significance of differences between different groups.

Interferons and interleukins are key downstream signaling effectors stimulated by PRRs in macrophages and dendritic cells (DCs). Specifically, IFNs mediate intracellular communication to trigger antiviral immunity and eradicate virus replication. In this study, IFN-α, IFN-β and IFN-γ were highly up-regulated in the thymus, kidney and liver, whereas those cytokines were selectively up-regulated in the blood, bursa of Fabricius and Harderian gland (Fig 3). The expression levels of IFN-α and IFN-β in the bursa of Fabricius following infection with DHAV-1 CH60 strains were more than 4-fold greater than those induced by DHAV-1 H strains (*P*<0.001). This situation was similar to the effect on IFN-β expression in the liver, lung and spleen (*P*<0.001) (Fig 3). Interleukins, mainly synthesized by helper CD4+T lymphocytes monocytes, macrophages and endothelial cells promoted the proliferation and differentiation of T and B lymphocytes. We determined that IL-2 and IL-4 were all highly up-regulated in tissues infected with both DHAV-1 CH60 and H strains (Fig 4). However, the expression levels of IL-1β and IL-6 in those tissues were selectively up-regulated. Specifically, IL-1β in the DHAV-1 H strain -infected kidney was the only up-regulated gene, whereas this gene in the DHAV-1 CH60 strain-infected Harderian gland and liver was highly expressed compared with its expression in the H strain-infected tissues.

### Alternative up-regulation of MHC and BAFF and strong elevation of CCL21 are induced by the CH60 and H strains of DHAV-1

**Fig 5:**
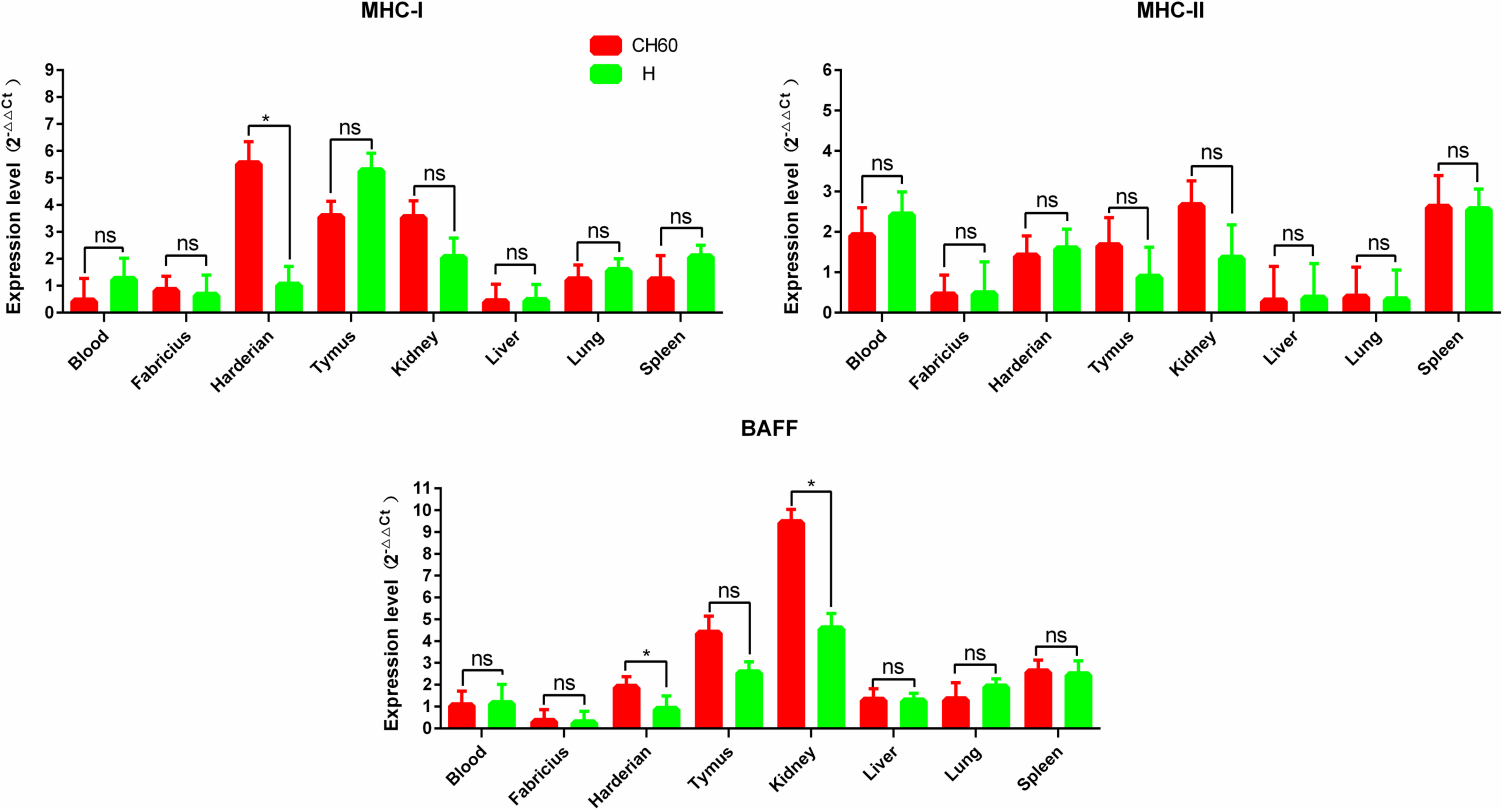
Expression patterns of MHC and BAFF in different organs. The relative expression levels of MHC-I and MHC-II were calculated using the 2^-ΔΔCt^ method. The error bars represent standard errors of the mean. Statistically significant differences between the mean gene expression levels in different organs were determined by one-way ANOVA (*P* < 0.05). *P < 0.05, **P < 0.01 or ***P < 0.001 indicate the level of statistical significance of differences between different groups.

**Fig 6:**
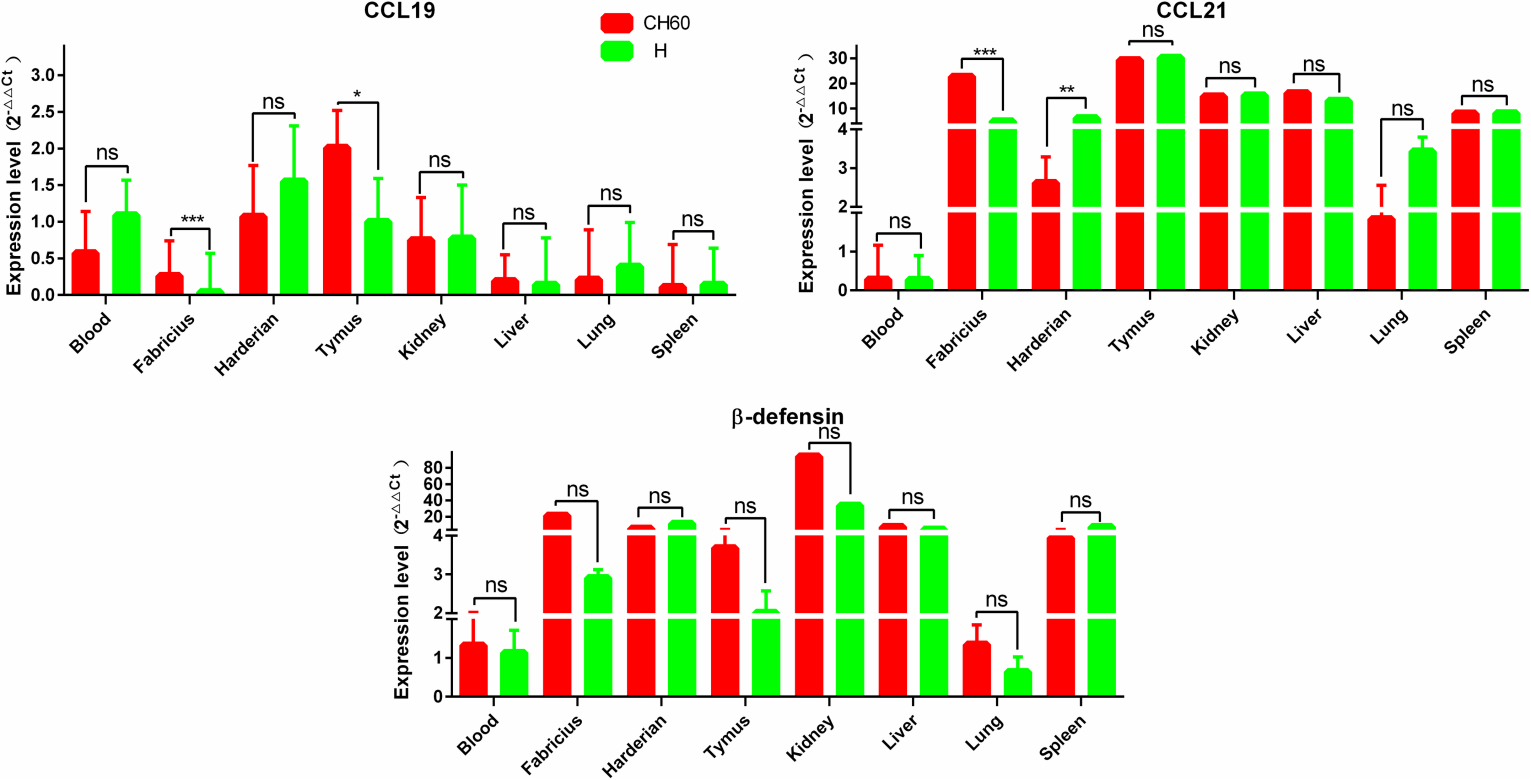
Expression patterns of chemokines and β-defensin in different organs. The relative expression levels of CCL19, CCL21 and β-defensin were calculated using the 2^-ΔΔCt^ method. The error bars represent the standard errors of the mean. Statistically significant differences between the mean gene expression levels in different organs were determined by one-way ANOVA (*P* < 0.05). *P < 0.05, **P < 0.01 or ***P < 0.001 indicate the level of statistical significance of differences between different groups.

During the early stages of the immune response, endogenous and exogenous antigens are presented on MHC-I and MHC-II molecules, respectively. Meanwhile, chemokines are also secreted to recruit lymphocytes to the sites of infection. MHC-I in thymus and MHC-II in spleen infected with both the CH60 and H strains were expressed at levels at least two-fold higher than those of the control groups (Fig 5). However, MHC-I was a selectively up-regulated gene in the Harderian gland infected with the DHAV-1 CH60 strain (Fig 5). Compared with the expression of MHC-I, the expression of MHC-II in major selected tissues was slightly and selectively up-regulated, specifically in the spleen and blood. BAFF was highly up-regulated in the spleen and thymus, which was essential for B cell survival. The expression level of CCL21 in all major tissues infected with either the CH60 or H strain, except for the blood, was more than 2-fold that of the control group (Fig 6). However, CCL19 expression levels in major tissues infected with the CH60 and H strains were comparatively lower than the expression levels of CCL21 and were only slightly up-regulated in the Harderian gland and thymus. Additionally, except in the blood and lung, β-defensin was also highly expressed in both CH60- and H strain-infected tissues.

### Higher nonstructural viral protein loads are associated with vacuolar degeneration in the liver and cytotoxic T cell responses

**Fig 7:**
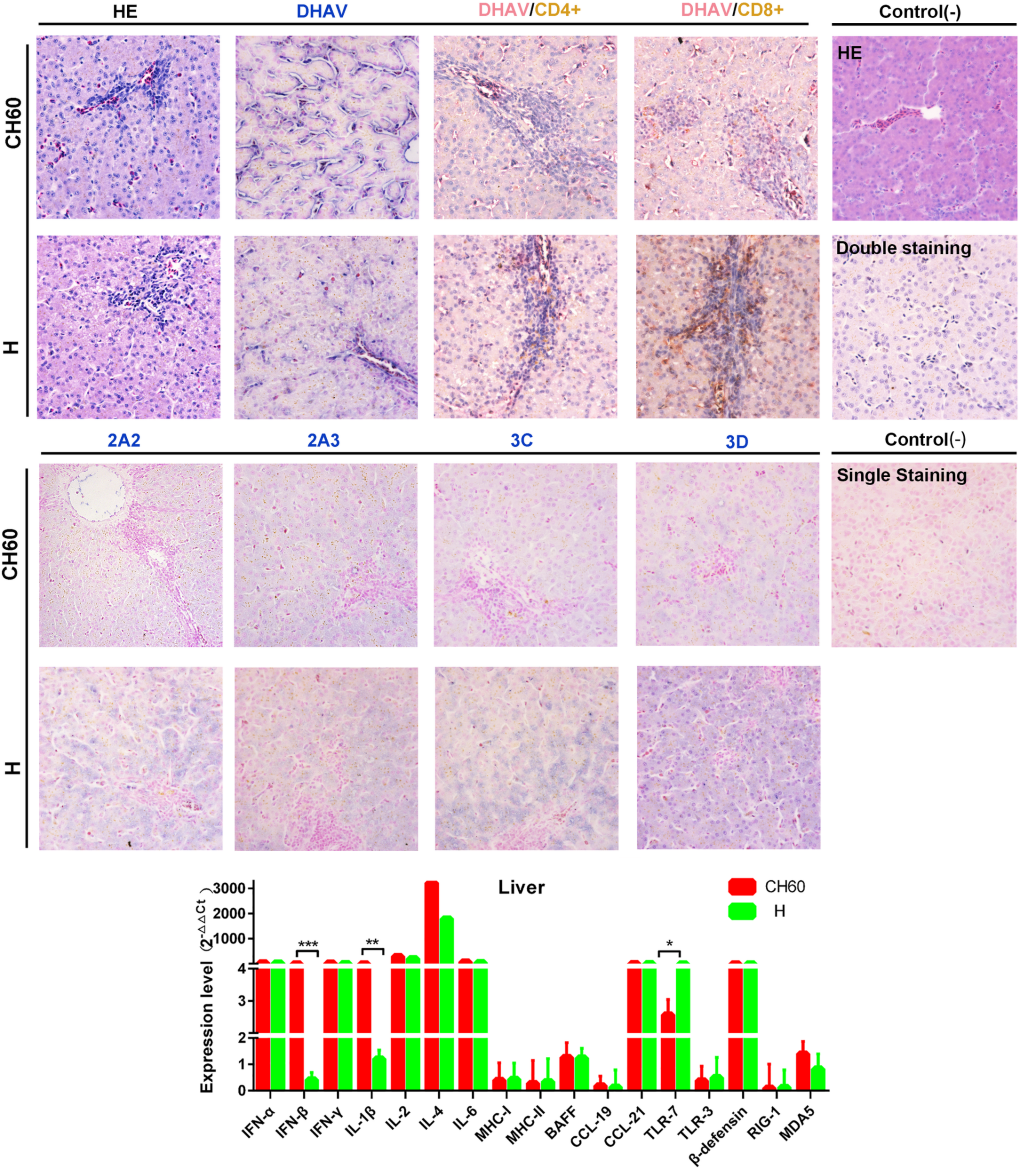
Comparative study of virus-host interactions mediated by attenuated and virulent strains in the liver. HE, single staining of the viral capsid, 2A2, 2A3, 3C and 3D and double staining of the viral capsid and CD4+ or CD8+ positive cells were performed to compare the pathological changes, viral protein expression levels and the extent of Th or Tc cell responses caused by the diversity of virulence. The positive staining of the viral capsid, 2A2, 2A3, 3C and 3D was visualized as bluish violet, while the double staining of the viral capsid and CD4+ or CD8+ was identified as red or brown, respectively. The negative control (-) refers to standard HE staining and single and double IHC staining without primary antibody. The relative expression levels of immune-related genes were also calculated using the 2^-ΔΔCt^ method. The error bars represent standard errors of the mean. Statistically significant differences induced by the attenuated and virulent strains between the mean gene expression levels were determined by one-way ANOVA. *P < 0.05, **P < 0.01 or ***P < 0.001 indicate the level of statistical significance of differences between different groups.

To identify the difference in immune responses induced by the virulent and attenuated strains, viral protein expression levels and virally induced CD4+ or CD8+ T cell immune responses were identified by single and double IHC staining. Routine HE staining was also used to investigate the histological changes. In the liver, vacuolar degeneration was apparently induced by virulent strains but not attenuated strains, especially in the circumference of the portal area (Fig 7). In addition, we also identified that the attenuated virion was limited in the area of the hepatic sinusoid, whereas the virulent virion infected most hepatic cells, especially in the circumference of the portal area. Additionally, CD8+ T cell-mediated immune responses were robustly induced by virulent strains compared with CD4+ T cell-mediated immune responses. The situation in liver tissues infected with attenuated strains was similar to a relatively weakened response (Fig 7). Additionally, comparison of immune-related genes in the liver induced by the CH60 and H strains revealed that interferon, interleukins, CCL21, TLR7 and β-defensin were expressed at levels almost 4-fold greater than those observed in the control group. However, compared with immune-related gene expression levels induced by H strains, IFN-β and IL-1β were significantly more highly expressed in tissues infected with CH60 strains (Fig 7). Intriguingly, TLR7 expression in liver infected with CH60 strains was lower than that infected with H strains.

### Strong cytotoxic T cell responses are induced by attenuated strains but not by virulent strains in the spleen

**Fig 8:**
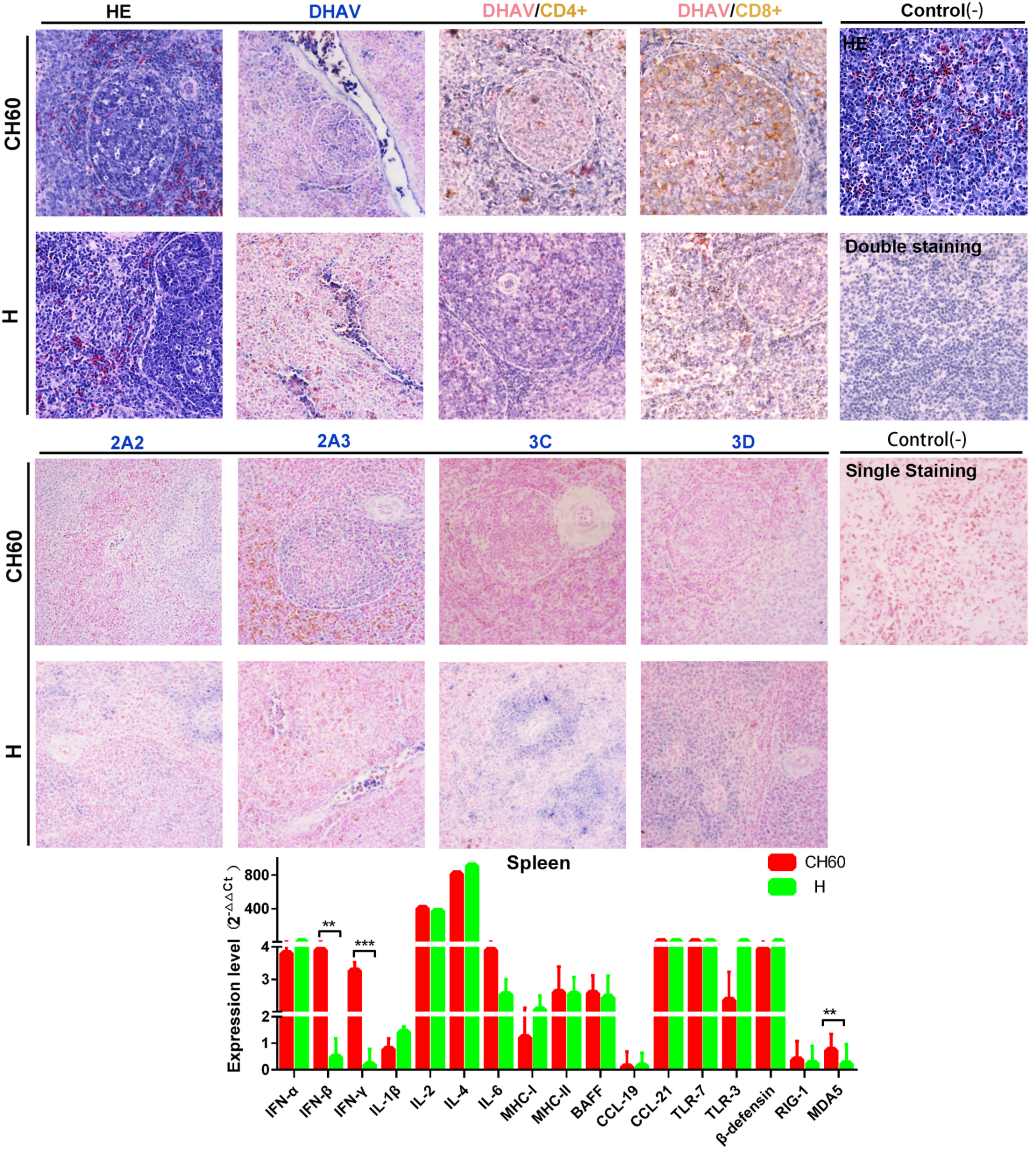
Comparative study of virus-host interactions mediated by attenuated and virulent strains in the spleen. HE, single staining of the viral capsid, 2A2, 2A3, 3C and 3D and double staining of the viral capsid and CD4+ or CD8+ positive cells was performed to compare the pathological changes, viral protein expression levels and the Th or Tc cell responses caused by the diversity of virulence. The positive staining of the viral capsid, 2A2, 2A3, 3C and 3D was visualized as bluish violet, while the double staining of the viral capsid and CD4+ or CD8+ was identified as red or brown, respectively. The negative control (-) refers to standard HE staining and single and double IHC staining without primary antibody. The relative expression levels of immune-related genes were calculated using the 2^-ΔΔCt^ method. The error bars represent standard errors of the mean. Statistically significant differences induced by attenuated and virulent strains between the mean gene expression levels were determined by one-way ANOVA. *P < 0.05, **P < 0.01 or ***P < 0.001 indicate the level of statistical significance of differences between the different groups.

Compared with the liver, the spleen is a key site of immunogenesis that can provide a crucial cytokine microenvironment for the development and proliferation of T and B lymphocytes. Splenic nodules can be formed in spleens infected with both virulent and attenuated DHAV-1 strains. However, the viral capsids of attenuated strains can be well presented at splenic nodules or trabecular veins, but to a relatively lower extent than that of the virulent strain-infected spleen (Fig 8). It is worth mentioning that the expression levels of nonstructural proteins in the virulent strain-infected spleen were highly limited at the periarterial lymphatic sheath. Similar to those observations, CD8+ T cell-mediated immune responses were comparatively higher that the CD4+ T cell-mediated immune responses in spleen tissues infected with either the attenuated or virulent strains. Those immune responses induced by the attenuated strains were apparently stronger than those observed in tissues infected with virulent strains (Fig 8). The patterns of highly expressed immune-related genes were similar to those observed in the liver except for the TLR3, BAFF, MHC-I, and MHC-II genes. Additionally, the expression levels of IFN-β, IFN-γ and MDA5 were significantly up-regulated by the attenuated strains compared with those of tissues infected with virulent strains (*P*<0.01 or *P*<0.001).

### Bounded versus scattered distribution without Th or Tc cell responses is mediated by attenuated and virulent strains in the kidney

**Fig 9:**
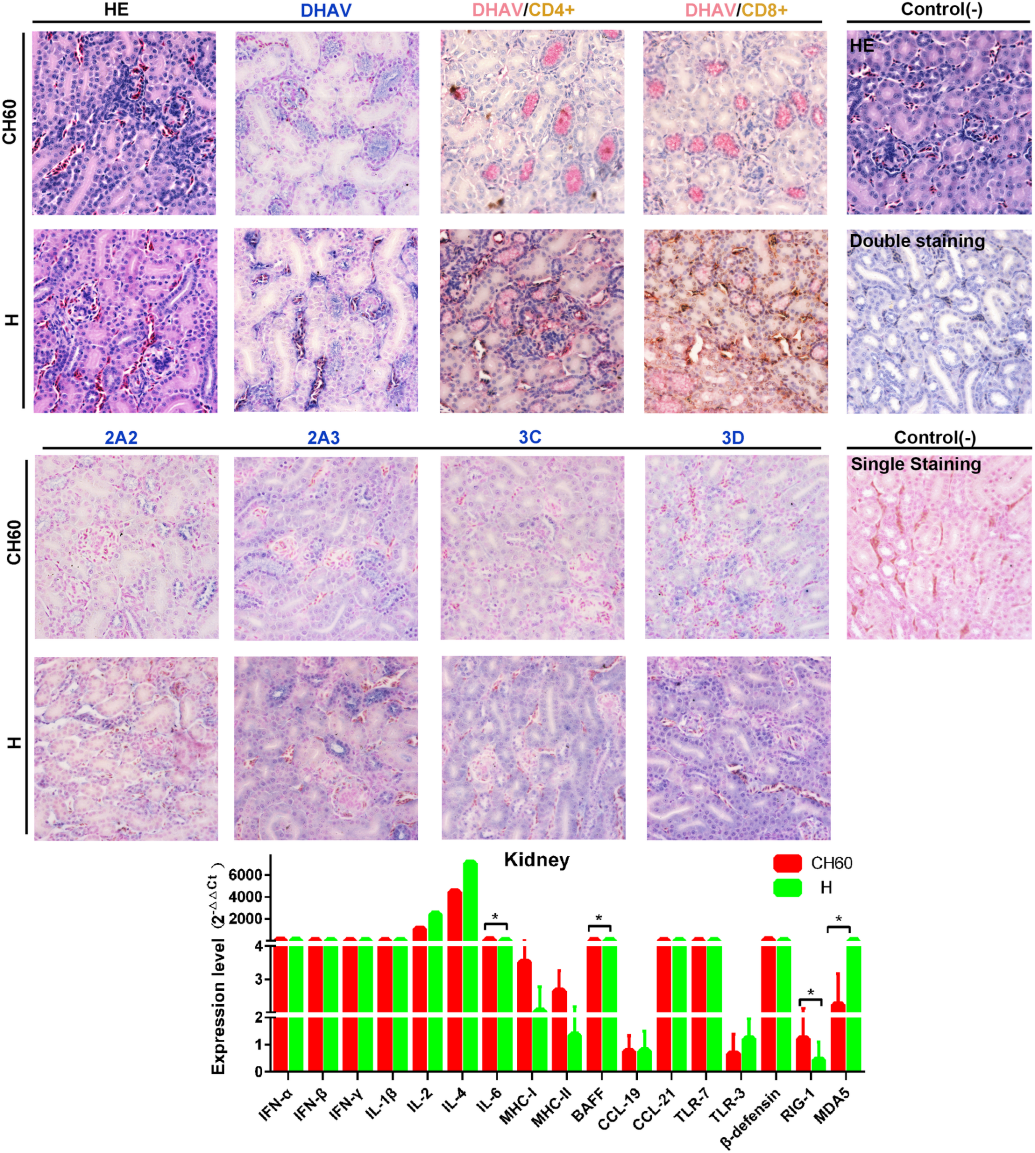
Comparative study of virus-host interactions mediated by attenuated and virulent strains in the kidney. HE, single staining of the viral capsid, 2A2, 2A3, 3C and 3D and double staining of the viral capsid and CD4+ or CD8+ positive cells were performed to compare the pathological changes, viral protein expression levels and the Th or Tc cell responses caused by the diversity of virulence. The positive staining of the viral capsid, 2A2, 2A3, 3C and 3D was visualized as bluish violet, while the double staining of the viral capsid and CD4+ or CD8+ was identified as red or brown, respectively. The negative control (-) refers to standard HE staining and single and double IHC staining without primary antibody. The relative expression levels of immune-related genes were calculated using the 2^-ΔΔCt^ method. The error bars represent the standard errors of the mean. Statistically significant differences induced by attenuated and virulent strains between the mean gene expression levels were determined by one-way ANOVA. *P < 0.05, **P < 0.01 or ***P < 0.001 indicate the level of statistical significance of differences between the different groups.

Due to the crucial function of physiological metabolism, the kidney plays an important role in homeostasis and is susceptible to many viral or bacterial infections. Some of the kidney tubular cells in virulent strain-infected kidney tissues, but not in the attenuated strain-infected kidneys, underwent acidophilic degeneration. Intriguingly, the attenuated virions were only located at the distal tubule, but the virulent virions infected the renal capsule, distal tubule and capillaries. In addition, the expression intensities of 3C and 3D proteins were higher than those observed in kidneys infected with attenuated strains (Fig 9). However, the CD4+ or CD8+ T cell immune responses were not identified in either the attenuated or virulent strain-infected kidneys, which is similar to our previous study [39]. The highly expressed interferon and interleukin genes in the kidney were also similar to those in the spleen and liver. BAFF, IL-6, RIG-1 and MDA5 were identified as differentially regulated genes following infection with attenuated or virulent strains in the kidney (*P*<0.05).

### Relatively lower loads of viral capsid and nonstructural proteins in both groups and strong Tc cell responses induced by virulent strains in the lung

**Fig 10:**
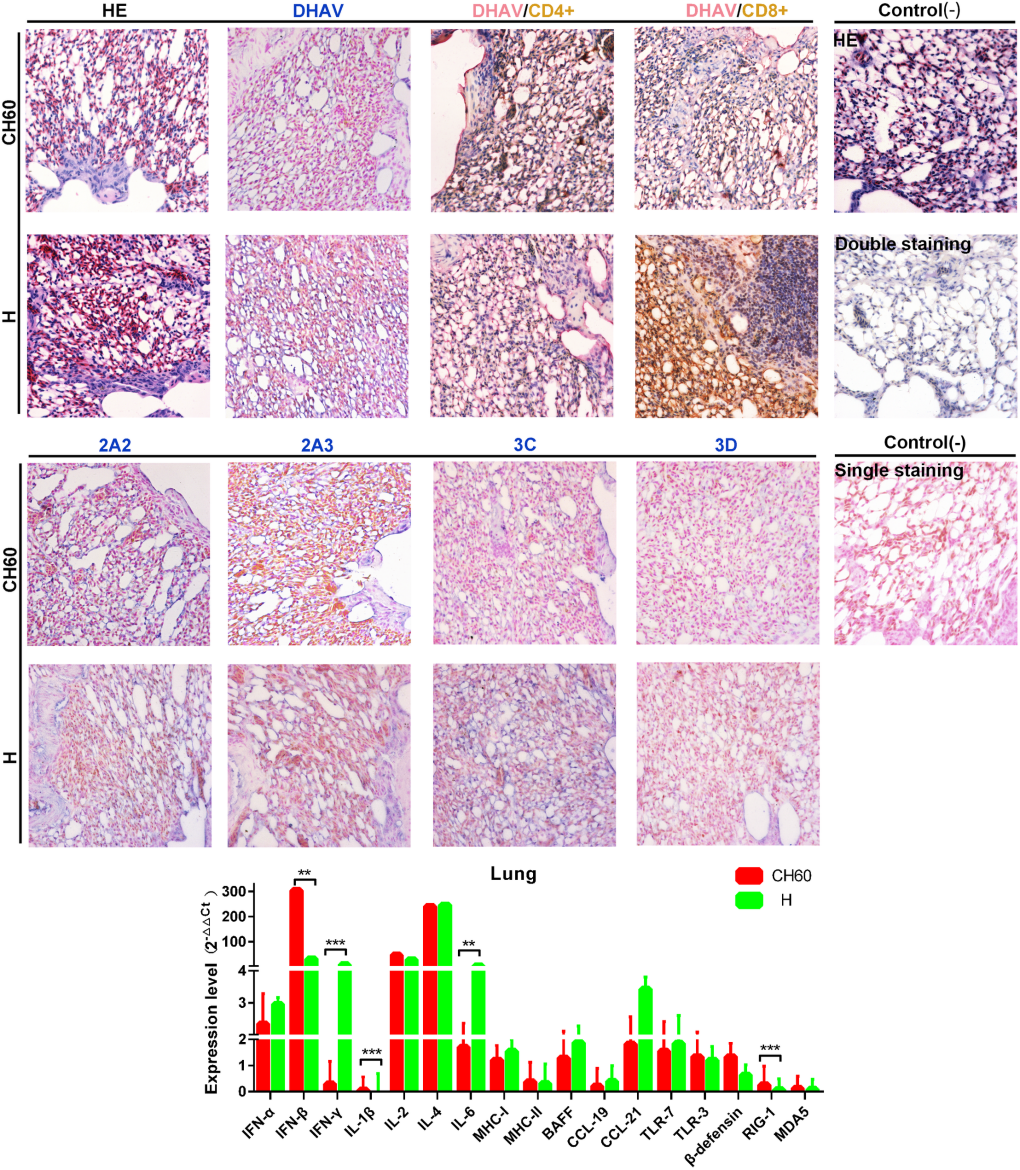
Comparative study of virus-host interactions mediated by attenuated and virulent strains in the lung. HE, single staining of the viral capsid, 2A2, 2A3, 3C and 3D and double staining of the viral capsid and CD4+ or CD8+ positive cells were performed to compare the pathological changes, viral protein expression levels and the Th or Tc cell responses caused by the diversity of virulence. The positive staining of the viral capsid, 2A2, 2A3, 3C and 3D was visualized as bluish violet, while the double staining of the viral capsid and CD4+ or CD8+ was identified as red or brown, respectively. The negative control (-) refers to standard HE staining and single and double IHC staining without primary antibody. The relative expression levels of immune-related genes were calculated using the 2^-ΔΔCt^ method. The error bars represent the standard errors of the mean. Statistically significant differences induced by attenuated and virulent strains between the mean gene expression levels were determined by one-way ANOVA. *P < 0.05, **P < 0.01 or ***P < 0.001 indicate the level of statistical significance of differences between the different groups.

The lung is also susceptible to many viruses and the site of mucosal immune response induction. No visible differences were observed between lung tissues infected with either the virulent or attenuated strains. The expression levels of DHAV capsid and nonstructural proteins (2A2, 2A3, 3C and 3D proteins) between those two groups were almost identical (Fig 10). However, CD8+ T cell-mediated immune responses were only induced by virulent strains in the infected lung tissues. In addition, the immune related genes RIG-1, IFN-β, IFN-γ, IL-1β and IL-6 were differentially regulated by CH60 or H strain infection in the lung (*P*<0.001 or *P*<0.01).

### Strong Th and Tc cell responses are induced by H strain with greater viral capsid and nonstructural protein loads in the Harderian gland

**Fig 11:**
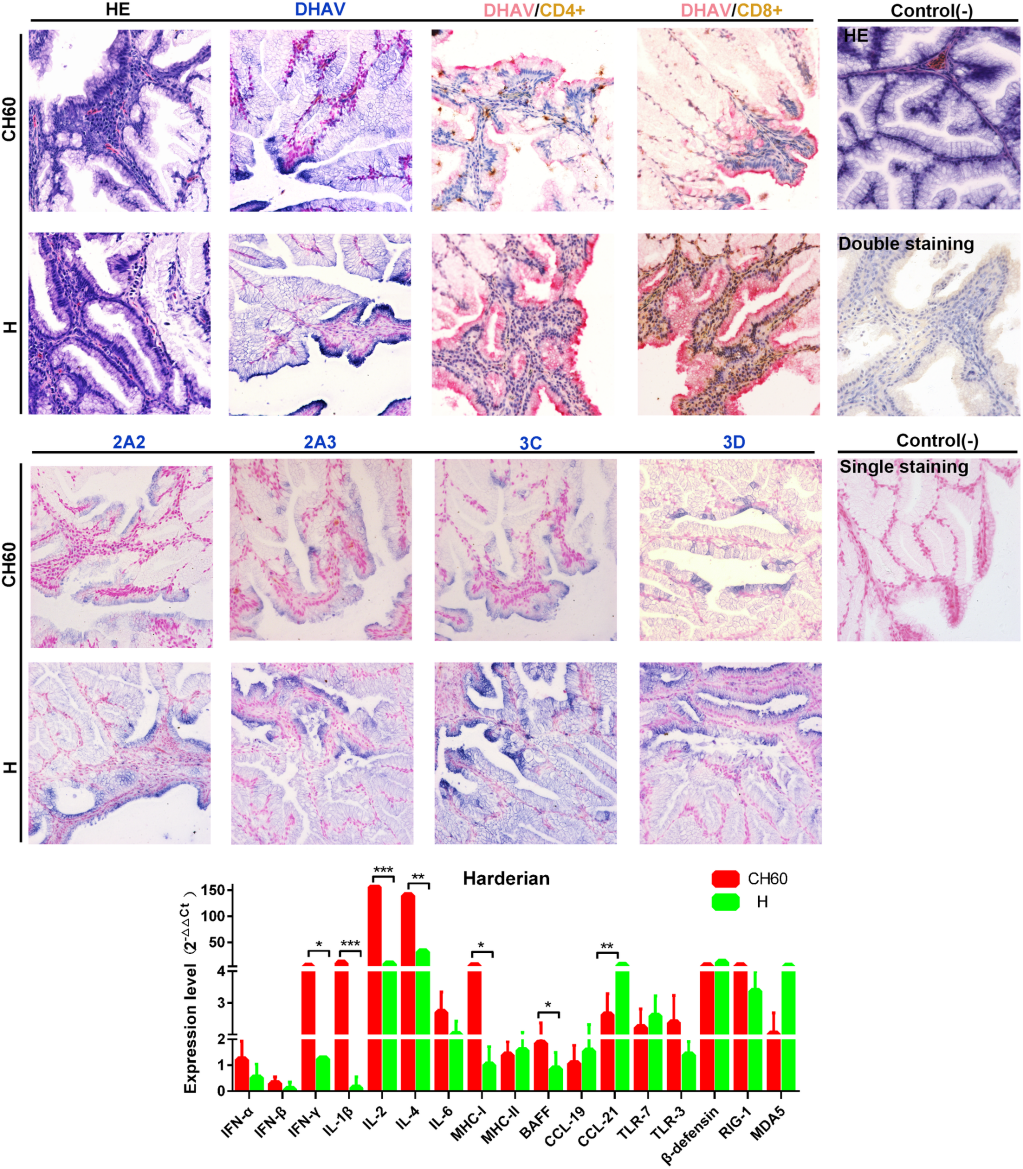
Comparative study of virus-host interactions mediated by attenuated and virulent strains in the Harderian gland. HE, single staining of the viral capsid, 2A2, 2A3, 3C and 3D and double staining of the viral capsid and CD4+ or CD8+ positive cells were performed to compare the pathological changes, viral protein expression levels and the Th or Tc cell responses caused by the diversity of virulence. The positive staining of the viral capsid, 2A2, 2A3, 3C and 3D was visualized as bluish violet, while the double staining of the viral capsid and CD4+ or CD8+ was identified as red or brown, respectively. The negative control (-) refers to standard HE staining and single and double IHC staining without primary antibody. The relative expression levels of immune-related genes were calculated using the 2^-ΔΔCt^ method. The error bars represent the standard errors of the mean. Statistically significant differences induced by attenuated and virulent strains between the mean gene expression levels were determined by one-way ANOVA. *P < 0.05, **P < 0.01 or ***P < 0.001 indicate the level of statistical significance of differences between the different groups.

The Harderian gland is a distinct avian immune organ that has a significant impact on the mucosal immune response. The Harderian gland is the only organ to undergo vacuolar degeneration in addition to the liver. The expression levels of DHAV capsid and nonstructural proteins (2A2, 2A3, 3C and 3D proteins) in the H strain-infected groups were comparatively lower than those in the attenuated strain-infected groups (Fig 10). In addition, robust CD8+ cytotoxic T cell responses and CD4+ Th cell responses were both induced by H strain, but weaker cellular responses were induced by the CH60 strains. IFN-γ, IL-1β, IL-2, IL-4, MHC-I, BAFF, and CCL21 expression levels in the Harderian gland were differentially regulated between the two groups (Fig 11).

### Strong elevation of the Tc cellular response is induced in both groups with higher expression of nonstructural viral proteins in the H strain infected thymus

**Fig 12:**
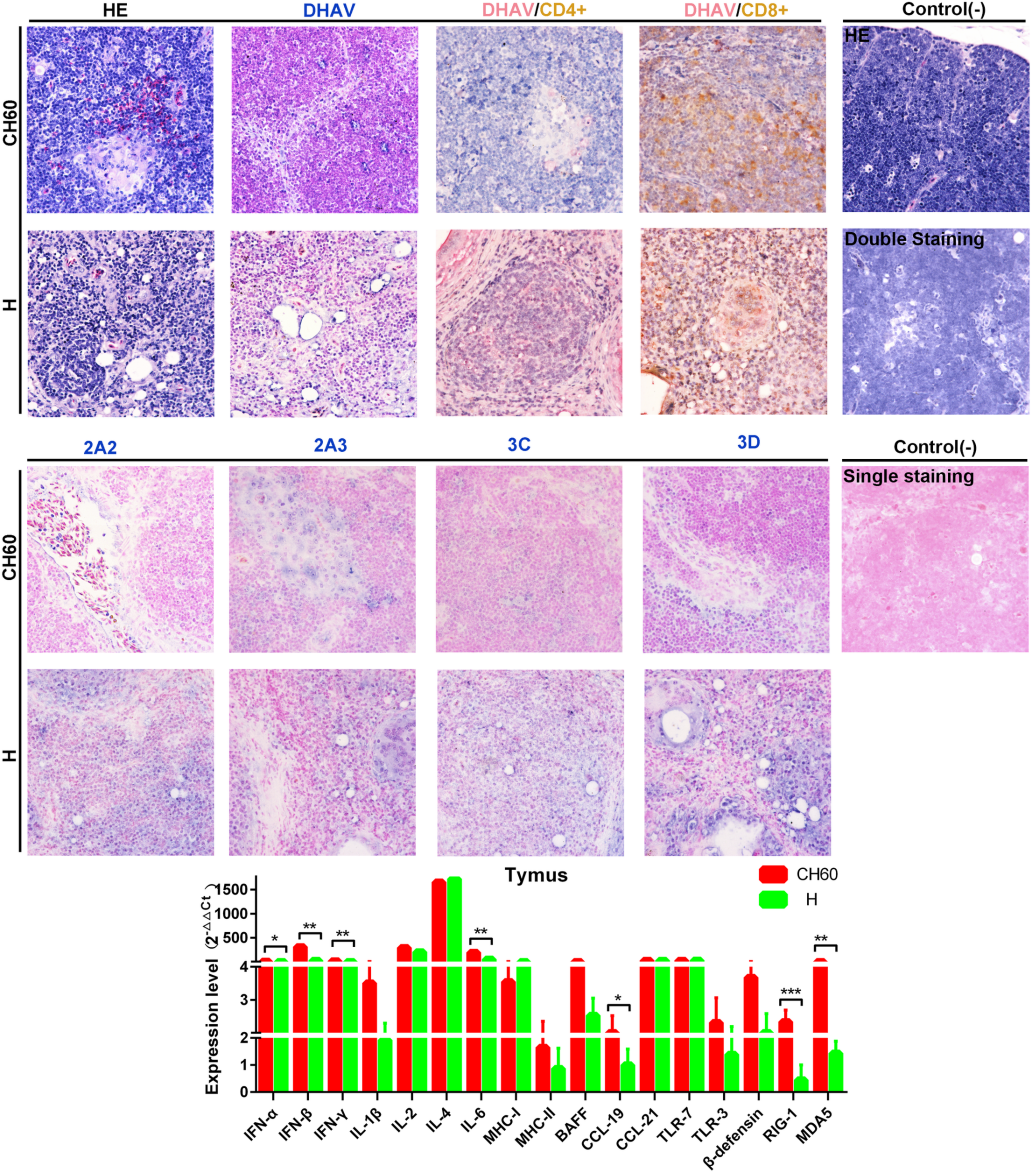
Comparative study of virus-host interactions mediated by attenuated and virulent strains in the thymus. HE, single staining of the viral capsid, 2A2, 2A3, 3C and 3D and double staining of the viral capsid and CD4+ or CD8+ positive cells were performed to compare the pathological changes, viral protein expression levels and the Th or Tc cell responses caused by the diversity of virulence. The positive staining of the viral capsid, 2A2, 2A3, 3C and 3D was developed as bluish violet, while the double staining of the viral capsid and CD4+ or CD8+ was identified as red or brown, respectively. The negative control (-) refers to standard HE staining and single and double IHC staining without primary antibody. The relative expression levels of immune-related genes were calculated using the 2^-ΔΔCt^ method. The error bars represent the standard errors of the mean. Statistically significant differences induced by attenuated and virulent strains between the mean gene expression levels were determined by one-way ANOVA. *P < 0.05, **P < 0.01 or ***P < 0.001 indicate the level of statistical significance of differences between the different groups.

The thymus, a specialized primary lymphoid organ, provides an environment conducive to the development of T lymphocytes that is also critical for adaptive immunity. Relatively larger populations of heterophilic granulocytes were recruited to thymus tissues infected with attenuated strains than to those infected with virulent strains. Intriguingly, a strong elevation of DHAV capsid protein expression levels was identified in thymus tissues infected with attenuated strains, but the nonstructural proteins were expressed at relatively lower levels when compared with those of thymus tissues infected with virulent strains (Fig 12). In addition, the attenuated strains can also induce robust CD8+ cytotoxic T cell responses and weak CD4+ Th cell responses (Fig 12). The immune responses in virulent strain-infected thymus tissues were weaker than those observed in thymus tissues infected with attenuated strains. In addition, we also identified seven genes that were differentially regulated due to virulence, including IFN-α, IFN-β, IFN-γ, IL-6, CCL19, RIG-1 and MDA5 (*P*<0.05).

### Strong Th and Tc cell responses are induced by virulent strains with higher viral capsid and nonstructural protein loads in the bursa of Fabricius

**Fig 13:**
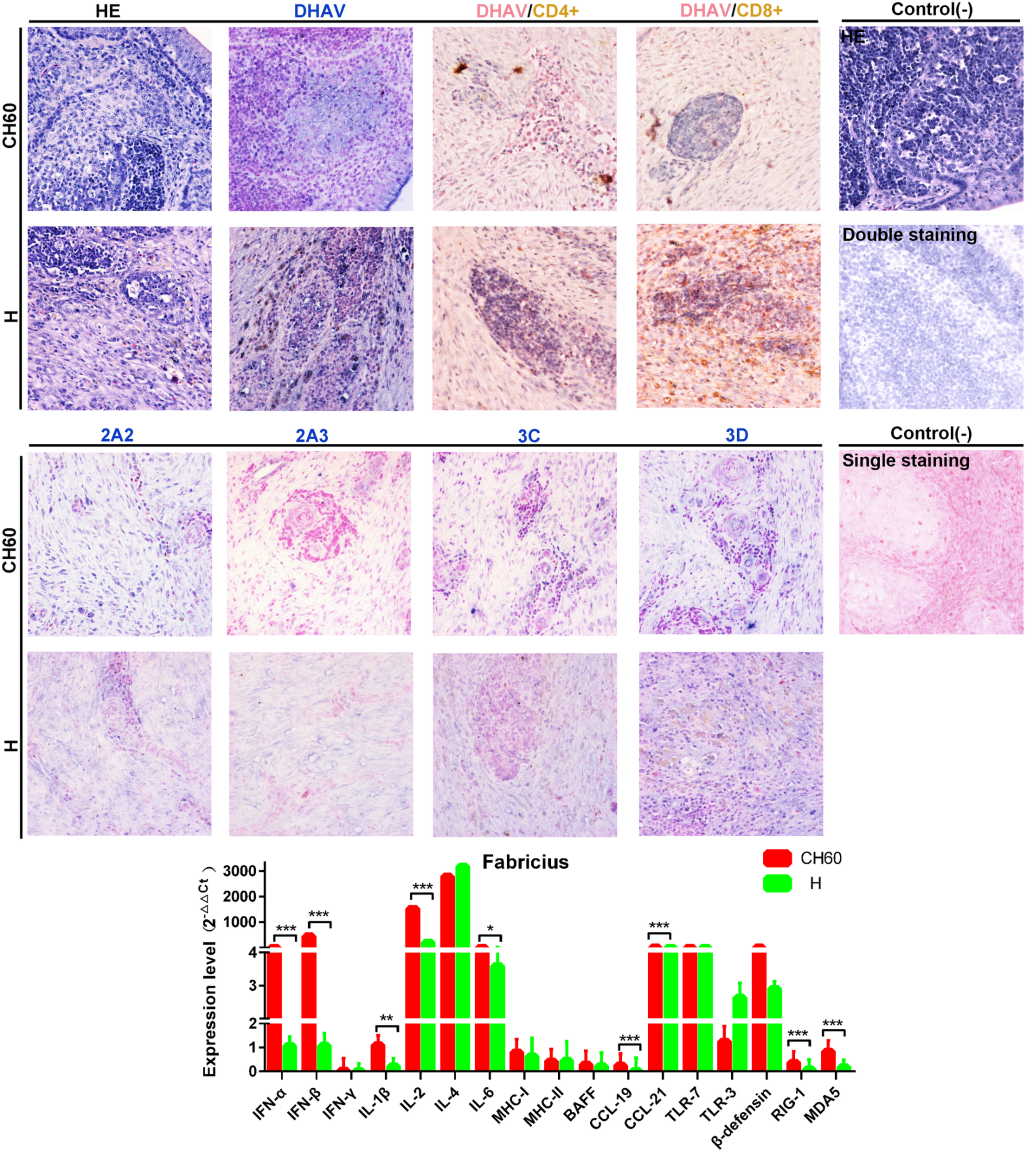
Comparative study of virus-host interactions mediated by attenuated and virulent strains in the bursa of Fabricius. HE, single staining of the viral capsid, 2A2, 2A3, 3C and 3D and double staining of the viral capsid and CD4+ or CD8+ positive cells were performed to compare the pathological changes, viral protein expression levels and the Th or Tc cell responses caused by the diversity of virulence. The positive staining of the viral capsid, 2A2, 2A3, 3C and 3D was visualized as bluish violet, while the double staining of the viral capsid and CD4+ or CD8+ was identified as red or brown, respectively. The negative control (-) refers to standard HE staining and single and double IHC staining without primary antibody. The relative expression levels of immune-related genes were calculated using the 2^-ΔΔCt^ method. The error bars represent the standard errors of the mean. Statistically significant differences induced by attenuated and virulent strains between the mean gene expression levels were determined by one-way ANOVA. *P < 0.05, **P < 0.01 or ***P < 0.001 indicate the level of statistical significance of differences between the different groups.

Due to the critical role of the bursa of Fabricius, it provides a specific environment for the differentiation and proliferation of B lymphocytes and is also critical to humoral immunity. No visible differences were observed in the bursa of Fabricius tissues between the two groups. The expression levels of capsid proteins were almost the same between the two groups, but the expression patterns of nonstructural proteins in the virulent strain-infected groups varied greatly in the bursa of Fabricius and were lower than the expression levels observed in the attenuated strain-infected groups (Fig 13). Additionally, the virulent strains induced a strong CD8+ cytotoxic T cell responses and a weaker CD4+ Th cell response. This interaction rarely occurred in the attenuated strain-infected groups. In addition, we also identified nine differentially regulated genes, including IFN-α, IFN-β, IL-1β, IL-2, IL-4, IL-6, CCL19, CCL21, RIG-1 and MDA5 (*P*<0.05) (Fig 13).

### Similar immune-related gene profiles induced by attenuated and virulent strains in the blood

**Fig 14:**
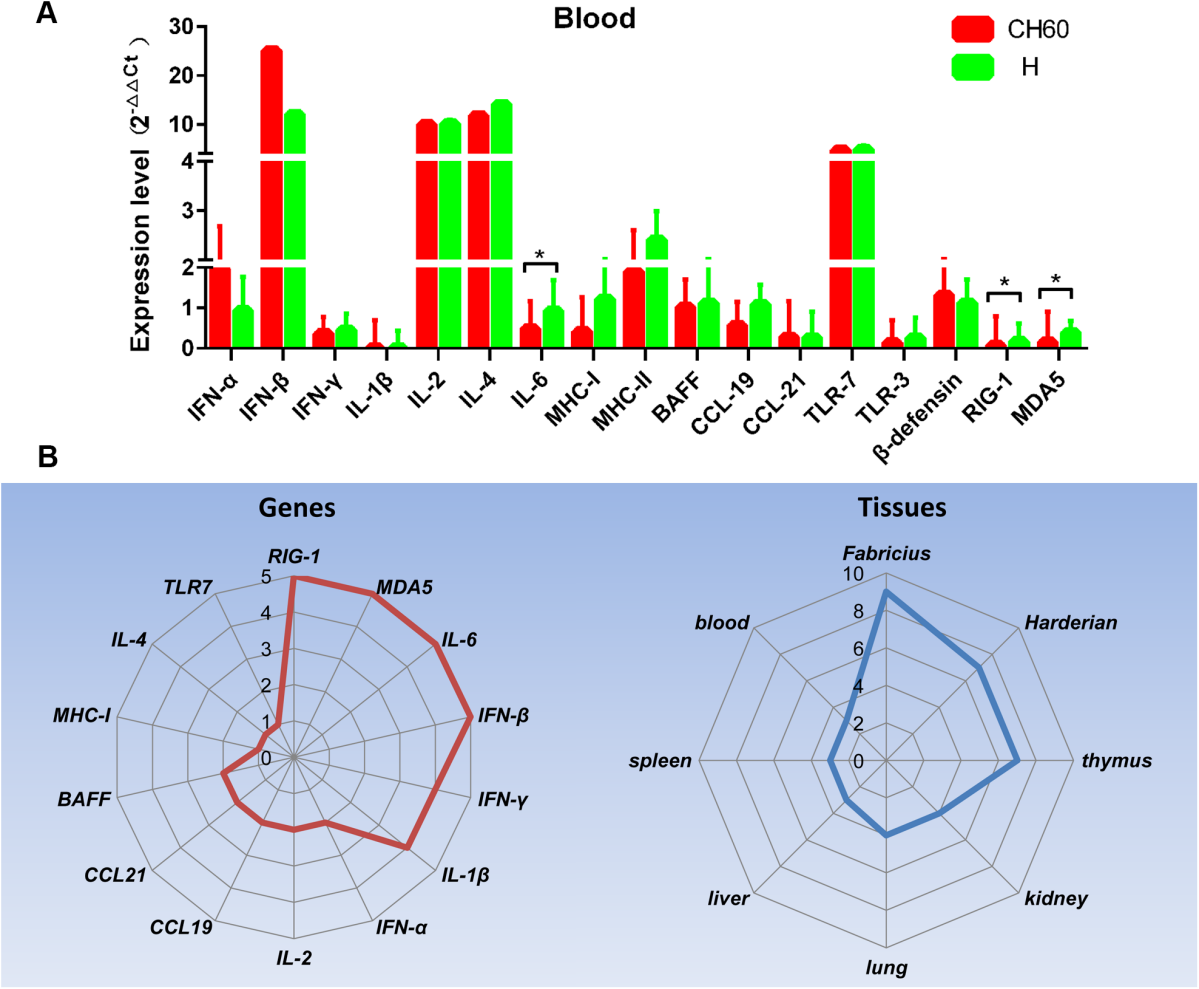
Overview of differentially regulated genes mediated by attenuated and virulent strains. A) The relative expression levels of immune-related genes in the blood were calculated using the 2^-ΔΔCt^ method. The error bars represent standard errors of the mean. Statistically significant differences induced by attenuated and virulent strains between the mean gene expression levels were determined by one-way ANOVA. *P < 0.05, **P < 0.01 or ***P < 0.001 indicate the level of statistical significance of differences between the different groups. B) The frequencies of differentially regulated genes in all selected tissues and specific tissues are shown as a Rader map (left and right).

The blood is frequently utilized by many pathogens as a conduit from the site of infection to the target organs and is also critical to lymphocyte migration. In this study, the expression levels of immune related genes in the blood were very similar between the two groups (Fig 14A). We determined that TLR7 was highly up-regulated in both groups as was IFN-β, IL-2 and IL-4. Three immune-related genes (RIG-1, MDA5 and IL-6) were identified as differentially regulated between the two groups in the blood (*P*<0.05) (Fig 14A).

## Discussion and conclusions

Live attenuated vaccines are widely used to protect humans or animals from certain pathogenic infections. Vaccine-induced immune responses are crucial to eradicating viral invasion. However, how virulence operates during the immune response is not entirely understood, at least in ducks. Seventeen immune-related genes expressed at different phases of immunogenesis, including 4 PRRs, 3 IFNs, 4 interleukins, 2 MHC, 2 chemokines, BAFF and β-defensin, activated CD4+ or CD8+ positive T cells and histological changes, were identified in this study. Viral capsid and nonstructural protein expression patterns were also investigated to determine the differential distribution caused by virulence.

In this study, the expression patterns of PRRs indicated that TLR7 expression was elevated in most selected tissues, whereas TLR3, RIG-1 and MDA5 were alternatively activated during the early immune response. This result was consistent with a previous study in which TLR7 was involved in the recognition of ssRNA, and the other three elements were recognized dsRNA [41, 42]. Activation of both types of PRRs by ssRNA or dsRNA-induced type I interferon and other inflammatory cytokine production [41, 42]. Consequently, highly up-regulated interferon (type I and II) and interleukins (IL-2 and IL-4) are induced by the CH60 and H strains of DHAV-1. Those cytokines may participate in the following cytokine storms for an early immune response as identified in the kidney [39]. Meanwhile, viral proteins were processed by the proteasome or endosome and then presented by the MHC-I or MHC-II molecules on APCs, which can further interact with CD4+ T cells and CD8+ T cells [29, 30]. Compared with the higher expression of MHC-I in the Harderian gland, thymus and kidney, slightly up-regulated MHC-II expression in the spleen and blood indicated that different tissues were involved in creating diverse immune strategies against the viral infection. In addition, chemokines are responsible for attracting T/B cells, APCs and other lymphocytes [31]. CCL21, but not CCL19, was significantly elevated in most selected tissues, and consistent with this finding, more neutrophils were recruited to the portal area of the liver [43]. Intriguingly, besides CCL21 up-regulation in both groups, CCL19 expression in thymus tissues infected with virulent strains was highly up-regulated within increased populations of heterophilic granulocytes [44]. These data suggested that CCL21 and CCL19 were important in the recruitment of heterophilic granulocytes in the thymus, which is consistent with the fact that larger and more organized infiltrates occur in mice with ectopic CCL21 expression [44, 45]. In addition, strong elevation of BAFF in the thymus and spleen indicated that the differentiation and proliferation of B cells occurred after 2 days of infection [32]. β-defensin, an antibacterial peptide with chemotactic and immunomodulatory activities produced by avian heterophiles, may be used as a molecular adjuvant for avian vaccines [46–48]. We found that transcription of this cytokine was markedly up-regulated in the bursa of Fabricius, Harderian gland, thymus, kidney, liver and spleen, which was similar to the transcriptome analysis previously conducted in ducks infected with Avian Influenza [49].

In this work, we also identified that higher loads of viral capsid and nonstructural proteins were apparent in all tissues infected with virulent strains, but they were weaker in tissues infected with attenuated strains. This situation may be caused by codon usage bias in the attenuated virus, given the diversity of the translation systems utilized by chickens and ducks. The higher expression levels were accompanied by apparent histological changes, such as vacuolar degeneration in the liver and Harderian gland and the presence of apoptotic cells in the kidney. Consequently, strong cytotoxic T cell responses were induced by the virulent strains, but comparatively weaker CD4+ T cell responses were observed in the attenuated strain-infected liver, lung, bursa of Fabricius and Harderian gland. However, in the spleen and thymus, significantly elevated cytotoxic T cell responses were induced by the attenuated strains, but not by the virulent strains. Intriguingly, no Tc or Th cell responses were observed in the kidney, and this may be a viral strategy used to escape host immunity [39]. These differential immune trends may be caused by differentially regulated immunogenesis genes. From the overview of all immune-regulated genes, RIG-1, MDA5, IFN-β, and IL-6 were identified as frequently differentially regulated genes between the two groups (Fig 14B). In addition, the Harderian gland, bursa of Fabricius and thymus were the three main tissues with frequently differentially regulated genes (Fig 14B).

**Fig 15:**
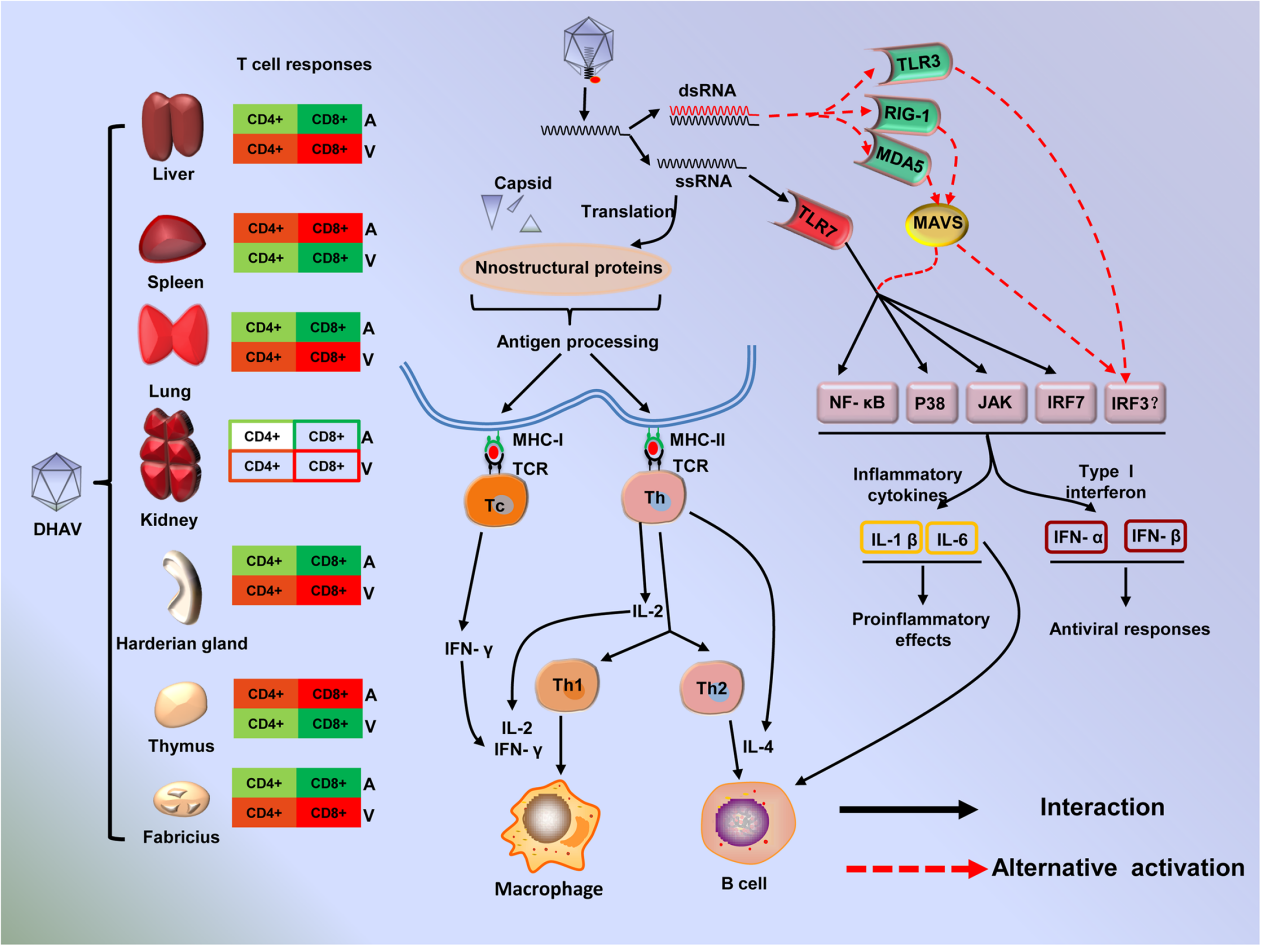
Overview of virus-host interactions induced by attenuated and virulent strains at early stages of the immune response [50]. Different trends in Tc or Th cell immune responses were induced by attenuated (A) and virulent (V) strains, and the red or green colors represent the major or minor trends of immune responses. The shade represents the level of each immune trend. Virus-associated ssRNA or dsRNA was recognized by TLR7 or alternatively activated TLR3, RIG-1 and MDA5, which are vital for the production of inflammatory cytokines and type I interferons through the NF-κB, IRF3/7 or MAPK signaling pathways. Meanwhile, the viral capsid and nonstructural proteins were translated in the host cell and presented by MHC-I or MHC-II on the surface of APCs. Then, CD8+ Tc or CD4+ Th cells were activated by antigen-MHC-I or antigen-MHC-II molecules, which were stimulated by IL-2, IL-4 and IFN-γ.

Collectively, virus-related molecular pattern recognition by the host PRRs is vital for the induction of the innate immune response to protect hosts from a viral infection. The viral intermediates, ssRNA and dsRNA, are generally recognized by TLR7 and TLR3; moreover, short dsRNA and long dsRNA can be recognized by RIG-1 and MDA5 [25, 26, 41] (Fig 15). The different types of ssRNA and dsRNA indicate a diversity of viral activity, translation and replication. Due to their lower translation efficacy and lower expression levels of viral proteins, the attenuated strains may produce low levels of replication intermediates (dsRNA). As expected, TLR7 was highly up-regulated with greater than 4-fold changes in most tissues, and TLR3, RIG-1 and MDA5 were selectively elevated in some tissues. Activation of those PRRs can induce the production of interferons and inflammatory cytokines through the NF-κB, MAPK or IRF3/7 signaling pathways [41] (Fig 15). These cytokines are vital for shaping the type of immune response, Th1 or Th2, which is essential for a successful outcome from a viral infection. Th1 cells triggered by IFN-γ, IL-2 and IL-12 are involved in cell-mediated immunity to maximize the killing efficacy of the macrophages and the proliferation of cytotoxic CD8+ T cells, whereas Th2 cells, triggered by IL-4, IL-5, IL-6, IL-9, IL-10 and IL-13, are involved in humoral immune response for B cell proliferation and class switching recombination [51, 52] (Fig 15). In this study, interferons (IFN-α, β, and γ) and interleukins (IL-2 and 4) were highly up-regulated in both groups. These data indicated that both cell-mediated immunity and humoral immune responses were shaped during early stages of the immune response. The double staining of DHAV capsid and CD4+ or CD8+ in the liver, lung, bursa of Fabricius and Harderian gland suggested that strong cytotoxic T cell responses were induced by virulent strains but were weaker in attenuated strains (Fig 15). These data suggested that CD8+ cytotoxic T cell responses were shaped, and those significantly elevated immune genes were involved in shifting to a Th1 cell response during the early stages of the immune response. In addition to the activation of T cells, the differentiation and proliferation were accompanied by a positive and negative selection of B cells, which is crucial for the production of antibody, immunoglobulin class switching and affinity maturation [32]. BAFF levels in the spleen and thymus were more up-regulated than in other tissues in both the attenuated and virulent groups, which is consistent with canonical immune responses. Additionally, peripheral NK cells, neutrophiles and heterophiles recruited to the sites of infection provide the necessary nonspecific immunity to enhance the function of cellular or humoral immunity. CCL21 was highly expressed in all tissues with much more heterophiles in the liver and thymus, which suggested that heterophiles may be recruited by CCL21 [44, 53]. This study provides vital information to clarify the details of virus-host interaction regulated by diverse virulence.

## Supporting information

S1 Table Representative viral strains in this analysis, with comments. (PDF)

S2 Table Primer sequences used in gene expression profiles. (PDF)

S3 Table RSCU of virulent strains and attenuated strains used in this study. (PDF)

S4 Table The original expression levels of immune-related genes as calculated by ΔCt (Cttarget-CtGAPDH). (PDF)
