# Supplementary material for: Comparative analysis of virus-host interactions caused by a virulent and an attenuated duck hepatitis A virus genotype 1
Source: PLoS One. 2017 Jun 14;12(6):e0178993. doi: 10.1371/journal.pone.0178993 (PMC5470708; doi:10.1371/journal.pone.0178993)
Supplement: S1 Table — (PDF) [file pone.0178993.s001.pdf]

S1 Table. Representative viral strains in this analysis, with comments.

Groups	Strains	Accession no.	Comments
Chicken embryo attenuated strains	CH60	KU923754.1	60 passages(china)
	C80	DQ864514	80 passages(china)
	FC64	HQ232302	64 passages(china)
	A66	DQ886445	66 passages(china)
	MY	JF914945	86 passages(china)
Virulent strains	LSD/090830	JF828989	China
	FZ99	JX390984	China
	NA	GQ130377	China
	C-XNH	GU066820	China
	ZJ	EU841005	China
	DRL-62	DQ219396	ATCC
	R85952	DQ226541	ATCC
	03D	DQ249299	Taiwan China
	JX	EU371557	China
	F	EU264072	China
	H	JQ301467	China
