# Supplementary material for: Comparative analysis of virus-host interactions caused by a virulent and an attenuated duck hepatitis A virus genotype 1
Source: PLoS One. 2017 Jun 14;12(6):e0178993. doi: 10.1371/journal.pone.0178993 (PMC5470708; doi:10.1371/journal.pone.0178993)
Supplement: S2 Table — (PDF) [file pone.0178993.s002.pdf]

S2 Table. Primer sequences used in gene expression profiles.

Gene	Forward primer	Reverse primer	Accession number	Reference
GAPDH	5'-ATGTTCGTGATGGGTGTGAA-3'	5'-CTGTCTTCGTGTGTGGCTGT-3'	AY436595	[38]
IFN-α	5'-TCCTCCAACACCTCTTCGAC-3'	5'-GGGCTGTAGGTGTGGTTCTG-3'	EF053034	[38]
IFN-β	5'-CCTCAACCAGATCCAGCATT-3'	5'- GGATGAGGCTGTGAGAGGAG-3'	AY831397	[38]
IFN-γ	5'-GCTGATGGCAATCCTGTTTT-3'	5'- GGATTTTCAAGCCAGTCAGC-3'	AJ012254	[38]
IL-1β	5'-TCGACATCAACCAGAAGTGC-3'	5'-GAGCTTGTAGCCCTTGATGC-3'	DQ393268	[38]
IL-2	5'-GCCAAGAGCTGACCAACTTC-3'	5'-ATCGCCCACACTAAGAGCAT-3'	AF294323	[38]
IL-4	5'-CCTCCACGGTTGTTTTCGAG-3'	5'-GTTGGAGGGTTCTGTGGAGG-3'	XM_005024359.1	[39]
IL-6	5'-TTCGACGAGGAGAAATGCTT-3'	5'-CCTTATCGTCGTTGCCAGAT-3'	AB191038	[38]
MHC-I	5'-GAAGGAAGAGACTTCATTGCCTTGG-3'	5'-CTCTCCTCTCCAGTACGTCCTTCC-3'	AB115246	[38]
MHC-II	5'-CCACCTTTACCAGCTTCGAG-3'	5'-CCGTTCTTCATCCAGGTGAT-3'	AY905539	[38]
BAFF	5'-TGTGCACGTCATCCAACAGA-3'	5'-GCCACAGGAATGTGACAGGA-3'	DQ445092	[39]
CCL21	5'-GGAGAAGCAGAAGAACCCCC-3'	5'-GGGAAAGCATCCGTCCTCTC-3'	DR764376	[39]
CCL19	5'-CCAGGAAGGTCCCAAATAAA-3'	5'-GTAGTAGGAGGTGGAAGCAAGTC-3'	DR766004	[39]
β-defensin	5'-CCAGGTTTCTCCAGGATTGT-3'	5'-AACCCAAAGCAACTTCCAAC-3'	AY641439	[39]
TLR7	5'-CCTTTCCCAGAGAGCATTCA-3'	5'-TCAAGAAATATCAAGATAATCACATCA -3'	AY940195	[38]
TLR3	5'-AACACTCCGCCTAAGTATCAT-3'	5'-CTATCCTCCACCCTTCAAAA-3'	JN573268	[39]
RIG-1	5'-GCGTACCGCTATAACCCACA-3'	5'-CCTTGCTGGTTTTGAACGC-3'	AB772012.1	[39]
MDA5	5'-GCTGAAGAAGGCCTGGACAT-3'	5'-TCCTCTGGACACGCTGAATG-3'	KJ451070.1	[39]
