# Supplementary material for: Comparative analysis of virus-host interactions caused by a virulent and an attenuated duck hepatitis A virus genotype 1
Source: PLoS One. 2017 Jun 14;12(6):e0178993. doi: 10.1371/journal.pone.0178993 (PMC5470708; doi:10.1371/journal.pone.0178993)
Supplement: S3 Table — (PDF) [file pone.0178993.s003.pdf]

S3 Table. RSCU of Virulent strains and attenuated strains used in this study.

Strains	Leu UUA	Leu CUA	Val GUA	Ser UCA	Thr ACA	Pro CCA	Ala GCA	Lys AAA	Gln CAA	Gly GGA	Arg AGA	Arg CGA	Glu GAA	IleA UA	Ser AGU	Asp GAU	Arg CGU
A66	0.67	0.64	0.57	1.85	1.67	2	1.42	1.16	1.09	1.07	1.87	0.68	1.17	0.62	0.94	1.27	0.45
C80	0.64	0.67	0.57	1.88	1.66	1.98	1.42	1.16	1.1	1.07	1.87	0.68	1.16	0.62	0.96	1.31	0.45
CH60	0.67	0.64	0.57	1.86	1.66	1.98	1.42	1.16	1.1	1.08	1.87	0.68	1.16	0.62	1.03	1.31	0.45
FC64	0.67	0.64	0.58	1.91	1.65	1.98	1.42	1.17	1.1	1.12	1.85	0.67	1.15	0.64	0.99	1.32	0.45
MY	0.67	0.64	0.57	1.85	1.66	1.98	1.42	1.17	1.1	1.08	1.92	0.68	1.16	0.62	1.06	1.3	0.45
3D	0.67	0.55	0.56	1.82	1.71	2.02	1.42	1.12	1.1	1.04	1.98	0.72	1.22	0.62	1.08	1.33	0.55
LSD/090830	0.75	0.51	0.5	1.78	1.64	1.96	1.4	1.11	1.04	1.09	1.96	0.71	1.2	0.64	1.12	1.27	0.44
C-XNH	0.58	0.77	0.46	1.78	1.64	1.91	1.45	1.1	1.07	1.03	1.98	0.61	1.19	0.64	1.18	1.34	0.5
FZ99	0.61	0.67	0.54	1.82	1.58	1.93	1.47	1.07	1.09	0.97	1.93	0.66	1.24	0.6	1.12	1.34	0.5
ZJ	0.58	0.64	0.57	1.92	1.65	1.96	1.38	1.1	1.04	0.95	1.96	0.65	1.25	0.6	1.08	1.33	0.49
NA	0.65	0.65	0.55	1.92	1.66	1.96	1.44	1.1	1.04	0.95	1.93	0.66	1.23	0.59	1.04	1.37	0.5

Strains	Gly GGU	Cys UGU	Asn AAU	Tyr UAU	His CAU	Ala GCU	Phe UUU	Leu CUU	Val GUU	Pro CCU	Thr ACU	IleA UU	Ser UCU	Gln CAG	Ala GCG	Arg CGG	Gly GGG
A66	1.01	1.33	1.37	1.47	1.18	1.51	1.35	1.38	1.35	1.26	1.5	1.69	1.89	0.91	0.09	0.68	0.91
C80	1.02	1.33	1.37	1.44	1.22	1.51	1.38	1.41	1.35	1.27	1.52	1.69	1.85	0.9	0.09	0.68	0.91
CH60	1	1.33	1.35	1.5	1.22	1.51	1.38	1.41	1.35	1.27	1.52	1.69	1.82	0.9	0.09	0.68	0.92
FC64	1.01	1.36	1.36	1.45	1.25	1.51	1.4	1.41	1.32	1.27	1.5	1.68	1.8	0.9	0.09	0.67	0.88
MY	1	1.33	1.35	1.53	1.22	1.48	1.4	1.41	1.35	1.27	1.52	1.71	1.81	0.9	0.12	0.68	0.92
3D	1.01	1.55	1.38	1.44	1.16	1.39	1.43	1.35	1.39	1.17	1.61	1.73	1.82	0.9	0.09	0.61	0.93
LSD/090830	1.09	1.48	1.35	1.46	1.35	1.49	1.41	1.44	1.41	1.26	1.56	1.88	1.74	0.96	0.18	0.6	0.85
C-XNH	1.05	1.43	1.4	1.53	1.18	1.39	1.35	1.35	1.46	1.17	1.52	1.75	1.71	0.93	0.09	0.66	0.95
FZ99	1.03	1.43	1.35	1.5	1.22	1.47	1.35	1.32	1.41	1.15	1.6	1.83	1.78	0.91	0.09	0.66	0.97
ZJ	0.95	1.55	1.39	1.5	1.18	1.47	1.4	1.49	1.39	1.26	1.6	1.83	1.85	0.96	0.15	0.65	1
NA	0.97	1.55	1.33	1.55	1.16	1.47	1.38	1.48	1.42	1.26	1.59	1.83	1.85	0.96	0.12	0.61	0.97

Strains	Glu GAG	Lys AAG	Thr ACG	Leu UUG	Ser UCG	Pro CCG	Val GUG	Leu CUG	Ala GCC	Asp GAC	Cys UGC	Arg CGC	Ser AGC	Arg AGG	Gly GGC	Tyr UAC	His CAC
A66	0.83	0.84	0.08	1.65	0.08	0.07	1.27	0.7	0.98	0.73	0.67	0.96	0.53	1.36	1.01	0.53	0.82
C80	0.84	0.84	0.07	1.62	0.08	0.07	1.27	0.73	0.98	0.69	0.67	0.96	0.54	1.36	0.99	0.56	0.78
CH60	0.84	0.84	0.07	1.65	0.08	0.07	1.27	0.7	0.98	0.69	0.67	0.96	0.53	1.36	1	0.5	0.78
FC64	0.85	0.83	0.08	1.65	0.08	0.07	1.29	0.7	0.98	0.68	0.64	1.01	0.54	1.35	0.99	0.55	0.75
MY	0.84	0.83	0.07	1.65	0.08	0.07	1.25	0.7	0.98	0.7	0.67	0.96	0.53	1.3	1	0.47	0.78
3D	0.78	0.88	0.08	1.68	0.12	0.07	1.27	0.77	1.1	0.67	0.45	0.94	0.39	1.21	1.01	0.56	0.84
LSD/090830	0.8	0.89	0.13	1.71	0.19	0.07	1.31	0.69	0.93	0.73	0.52	1.04	0.35	1.25	0.96	0.54	0.65
C-XNH	0.81	0.9	0.1	1.82	0.15	0.15	1.36	0.58	1.06	0.66	0.57	0.99	0.3	1.27	0.97	0.47	0.82
FZ99	0.76	0.93	0.1	1.78	0.15	0.15	1.33	0.67	0.96	0.66	0.57	0.99	0.35	1.27	1.03	0.5	0.78
ZJ	0.75	0.9	0.07	1.68	0.15	0.07	1.32	0.76	0.99	0.67	0.45	0.98	0.35	1.25	1.11	0.5	0.82
NA	0.77	0.9	0.07	1.69	0.12	0.07	1.34	0.71	0.97	0.63	0.45	0.99	0.38	1.32	1.11	0.45	0.84

Strains	Asn AAC	Phe UUC	Leu CUC	Ile AUC	Val GUC	Ser UCC	Pro CCC	Thr ACC
A66	0.63	0.65	0.95	0.69	0.81	0.72	0.67	0.75
C80	0.63	0.62	0.92	0.69	0.81	0.69	0.67	0.75
CH60	0.65	0.62	0.92	0.69	0.81	0.68	0.67	0.75
FC64	0.64	0.6	0.92	0.68	0.82	0.69	0.67	0.77
MY	0.65	0.6	0.92	0.67	0.83	0.68	0.67	0.75
3D	0.62	0.57	0.98	0.65	0.78	0.77	0.73	0.6
LSD/090830	0.65	0.59	0.9	0.48	0.78	0.81	0.7	0.68
C-XNH	0.6	0.65	0.89	0.61	0.72	0.87	0.77	0.75
FZ99	0.65	0.65	0.95	0.57	0.72	0.77	0.78	0.72
ZJ	0.61	0.6	0.85	0.57	0.72	0.65	0.7	0.67
NA	0.67	0.62	0.83	0.59	0.68	0.69	0.7	0.67
