# Supplementary material for: Comparative analysis of virus-host interactions caused by a virulent and an attenuated duck hepatitis A virus genotype 1
Source: PLoS One. 2017 Jun 14;12(6):e0178993. doi: 10.1371/journal.pone.0178993 (PMC5470708; doi:10.1371/journal.pone.0178993)
Supplement: S4 Table — (PDF) [file pone.0178993.s004.pdf]

S4 Table. The original expression levels of immune-related genes as calculated by ΔCt (Ct_{target}-Ct_{GAPDH}).

Control	IFN-α	IFN-β	IFN-γ	IL-1β	IL-2	IL-4	IL-6	MHC-I	MHC-II	BAFF	CCL-19	CCL-21	TLR-7	TLR-3	β-defensin	RIG-1	MDA5
Blood	7.08	17.57	16.82	8.68	16.03	12.30	16.06	-2.55	4.48	9.36	14.65	7.58	12.40	4.12	7.60	3.80	2.07
Blood	5.79	22.54	15.28	9.12	16.95	11.20	15.30	-2.42	4.51	9.87	13.79	7.25	13.48	5.17	7.02	4.37	1.65
Blood	6.05	18.99	17.39	7.45	16.99	12.10	15.16	-2.67	3.73	9.42	13.15	8.39	12.84	4.80	7.89	4.12	1.46
Blood	5.30	18.34	17.16	8.53	17.12	13.83	15.63	-2.68	4.09	9.42	13.49	7.38	13.31	4.56	8.30	4.33	1.84
Blood	7.33	20.55	17.01	8.64	17.56	12.33	15.41	-1.30	4.70	9.34	14.11	7.98	12.71	4.43	7.53	4.62	2.41
Fabricius	12.11	22.71	9.49	none	19.18	13.50	14.90	0.25	1.05	7.61	6.59	4.77	10.43	7.78	12.61	5.38	4.75
Fabricius	11.90	22.60	none	11.88	20.34	15.16	13.31	0.42	0.46	8.35	6.35	3.97	9.90	7.06	12.07	5.19	3.96
Fabricius	12.12	none	8.74	12.68	21.61	15.00	15.55	0.19	0.22	8.19	5.55	4.30	10.48	7.12	14.31	5.24	3.02
Fabricius	11.98	21.50	6.13	12.10	21.24	15.74	14.37	0.52	1.22	7.55	5.62	3.15	9.41	7.85	14.58	5.74	3.65
Fabricius	11.98	none	7.93	12.11	none	13.42	14.25	0.45	1.03	8.42	none	4.50	10.41	7.06	11.87	6.03	4.23
Hardrain	11.35	none	15.00	16.19	16.02	11.04	7.12	1.55	5.58	11.41	9.49	4.67	12.84	3.95	10.62	6.55	6.26
Hardrain	12.39	22.72	16.42	15.82	14.23	11.05	7.60	1.88	6.53	11.36	8.87	4.58	13.15	4.22	12.24	6.76	7.78
Hardrain	13.33	21.51	16.32	16.58	14.85	10.56	7.34	1.96	6.20	11.41	9.45	5.13	12.36	4.19	12.73	5.06	7.67
Hardrain	11.68	24.48	16.22	16.82	16.80	11.02	7.34	2.39	5.40	12.43	9.75	3.62	12.64	3.85	13.34	6.79	6.66
Hardrain	12.98	15.88	16.09	14.72	16.89	10.81	7.44	2.96	5.20	11.91	8.24	4.70	12.57	4.27	13.47	5.81	8.13
Kidney	14.93	24.56	15.89	17.00	23.86	17.00	20.49	2.97	7.73	15.33	11.35	7.32	16.97	5.59	14.74	9.31	6.90
Kidney	15.39	27.32	16.77	17.10	22.69	17.01	21.13	3.74	5.86	14.52	10.00	6.85	16.98	5.27	16.09	8.95	8.17
Kidney	14.92	25.22	16.57	17.11	21.45	16.39	20.41	3.97	6.17	15.57	11.83	6.92	16.71	5.06	14.51	9.59	8.66
Kidney	15.80	22.56	18.00	17.69	23.37	16.88	20.78	2.67	7.07	16.53	11.52	6.31	16.69	5.77	15.28	9.87	8.62
Kidney	14.91	20.76	18.71	17.76	23.27	15.57	20.71	2.23	7.24	16.66	11.37	7.40	16.70	5.70	13.44	9.53	7.50
Liver	13.45	23.87	14.49	13.00	20.80	14.20	18.98	0.56	3.03	11.76	7.76	4.01	14.60	5.27	9.82	5.36	7.82
Liver	13.53	22.29	15.29	14.44	20.52	15.77	18.43	0.83	2.66	12.81	6.57	4.40	14.37	5.28	10.88	6.25	6.52
Liver	13.98	24.57	15.87	14.76	21.09	14.67	19.36	0.51	2.83	11.95	6.71	4.80	14.57	3.97	10.19	6.74	6.65
Liver	14.74	23.02	17.87	15.58	20.23	15.15	19.13	0.72	3.28	12.57	5.11	4.75	14.22	4.82	8.24	5.36	6.93
Liver	14.38	21.55	16.78	13.12	21.06	15.19	19.47	0.23	3.92	12.46	5.76	4.45	15.12	4.62	8.77	5.86	5.33
Lung	10.54	21.13	15.75	8.32	17.71	11.40	13.40	-1.66	1.84	10.36	6.72	1.32	11.07	4.66	5.42	4.50	4.37
Lung	10.67	23.75	16.60	8.72	17.89	11.12	13.19	-1.08	1.09	9.82	6.17	0.53	11.29	3.90	5.72	4.64	5.54
Lung	8.44	19.76	15.48	6.84	17.66	11.44	13.42	-1.89	1.14	10.11	6.86	0.52	11.17	2.56	6.22	4.29	6.94
Lung	8.63	23.70	17.90	8.41	17.95	11.60	13.22	-2.07	1.12	10.09	5.35	1.15	11.38	4.89	5.31	4.76	4.14
Lung	10.16	21.78	17.40	7.88	17.51	10.78	13.85	-2.28	1.96	10.17	5.80	1.13	12.65	3.26	5.43	5.68	4.30
Spleen	12.17	18.07	6.21	12.77	19.29	15.23	12.66	-0.67	0.18	7.24	2.32	0.95	10.27	5.43	6.25	4.87	3.79
Spleen	11.66	22.09	8.40	12.77	21.49	14.71	13.20	-1.09	0.05	7.85	2.00	0.93	10.34	4.49	4.53	4.25	3.34
Spleen	11.01	21.79	6.23	13.69	19.80	14.25	12.49	-0.84	0.01	7.40	2.49	0.61	10.29	4.23	5.78	3.71	3.13
Spleen	10.85	21.62	7.55	12.97	21.72	15.09	14.05	-0.53	0.63	8.62	2.80	0.33	11.20	3.82	4.69	3.87	2.91
Spleen	11.52	21.67	8.74	13.83	20.61	14.05	13.99	-0.26	0.55	8.26	1.79	0.30	10.37	5.42	3.15	4.33	4.19
Tymus	11.54	25.92	14.85	11.81	18.28	13.80	17.32	none	2.52	9.26	6.44	2.50	12.89	4.33	3.18	7.13	4.68
Tymus	11.60	23.45	15.47	11.75	19.05	13.58	17.92	1.98	2.11	10.00	6.51	3.53	13.62	5.02	3.61	7.93	5.75
Tymus	11.81	21.66	15.10	13.02	19.91	12.47	19.30	2.20	1.46	10.71	7.29	3.51	13.70	4.52	5.34	9.14	6.19
Tymus	11.54	26.22	12.52	11.74	19.58	13.80	19.18	1.80	1.84	10.96	7.94	4.22	13.68	4.67	4.98	8.20	5.07
Tymus	11.15	24.05	16.67	12.57	19.49	14.18	16.90	2.07	2.32	10.04	6.93	3.07	13.26	5.74	4.19	7.84	5.34

CH60	IFN-α	IFN-β	IFN-γ	IL-1β	IL-2	IL-4	IL-6	MHC-I	MHC-II	BAFF	CCL-19	CCL-21	TLR-7	TLR-3	β-defensin	RIG-1	MDA5
Blood	6.47	18.53	17.91	14.11	15.48	8.80	17.04	-1.55	3.88	9.93	15.93	9.49	10.92	8.47	7.04	7.97	4.05
Blood	6.32	15.19	18.62	16.41	13.25	7.79	15.94	-2.19	2.85	9.24	16.07	10.48	11.12	7.47	7.76	8.50	18.39
Blood	6.77	14.73	24.66	15.74	13.16	6.67	16.54	-1.42	2.92	9.12	14.12	9.91	11.56	6.47	6.53	7.71	4.58
Blood	5.97	18.33	20.14	17.27	16.59	8.99	16.86	-1.02	3.76	9.94	15.65	12.62	12.24	8.17	7.36	8.48	4.32
Blood	5.17	15.44	18.01	16.86	14.03	8.28	16.35	-4.69	1.16	9.01	14.52	9.65	10.96	6.45	6.71	7.37	2.68
Fabricius	8.73	18.24	13.56	12.38	10.59	3.56	11.75	0.17	3.16	10.84	7.93	-0.12	9.37	3.69	9.80	6.50	6.35
Fabricius	9.02	13.80	13.28	12.45	10.24	4.35	12.63	1.03	1.73	9.73	12.23	1.92	7.87	7.32	12.03	7.67	5.15
Fabricius	8.85	23.08	13.51	11.30	10.66	3.40	11.35	0.83	2.04	10.05	6.95	-1.26	7.99	6.23	6.33	6.21	5.87
Fabricius	7.50	12.24	12.71	10.97	7.76	1.02	11.04	2.21	5.50	8.70	9.11	-0.20	9.64	4.66	7.16	8.99	9.86
Fabricius	8.73	14.29	12.46	14.00	10.43	4.11	11.37	-1.48	0.90	9.95	11.25	1.93	7.29	7.56	11.19	7.02	4.35
Hardrain	11.27	21.67	10.09	9.41	8.79	0.14	5.97	-0.31	6.89	9.31	7.02	-0.64	9.08	2.88	7.30	4.13	5.88
Hardrain	13.18	14.70	13.51	13.51	8.47	3.77	5.79	1.46	5.50	10.15	8.49	2.66	10.17	2.82	12.27	2.86	4.27
Hardrain	13.34	22.57	15.68	20.34	14.61	6.79	7.06	1.87	4.30	11.27	9.52	3.66	12.19	4.62	10.48	5.14	6.86
Hardrain	13.49	19.77	23.35	13.91	12.43	4.14	5.71	-0.50	6.84	10.44	9.38	2.96	11.82	2.87	10.73	3.82	6.84
Hardrain	12.37	16.75	13.31	11.81	10.60	3.65	6.19	-0.35	5.84	10.61	9.36	3.77	11.64	3.21	10.29	5.20	7.25
Kidney	8.31	20.22	15.45	16.01	13.46	6.05	14.65	2.70	6.31	13.49	11.47	3.09	13.96	6.13	9.19	9.45	7.37
Kidney	9.03	14.60	14.66	12.27	10.51	5.22	13.86	2.20	5.75	13.57	11.93	2.11	16.64	4.87	7.28	9.57	7.28
Kidney	9.34	18.95	14.13	14.78	none	5.72	15.32	1.66	4.99	12.63	9.67	2.44	11.05	4.68	-3.61	9.26	7.27
Kidney	9.40	19.39	23.49	13.92	14.69	4.31	13.98	3.01	8.06	12.12	11.88	3.19	13.27	6.25	8.41	10.67	9.40
Kidney	9.22	12.06	12.99	15.74	12.24	4.22	13.26	1.23	6.76	14.05	12.44	2.42	15.10	6.66	10.70	10.37	9.12
Liver	8.49	14.83	9.60	11.68	12.89	3.83	11.61	2.88	4.62	12.97	9.12	-0.24	11.55	5.75	7.68	8.27	6.01
Liver	9.36	20.53	9.90	11.39	12.58	2.02	13.87	2.13	3.89	11.16	9.04	0.23	14.39	6.70	7.28	9.58	6.64
Liver	8.40	none	11.20	12.25	12.13	3.39	12.02	1.87	5.01	13.27	10.70	0.35	12.67	6.53	7.32	9.59	5.77
Liver	9.97	21.13	12.41	13.30	13.54	3.10	12.39	1.89	5.08	11.61	11.24	0.79	15.33	6.07	6.10	9.35	6.30
Liver	8.80	18.59	10.78	13.60	12.55	4.41	14.67	1.03	4.65	11.48	10.22	2.00	12.03	5.87	8.08	9.29	6.63
Lung	9.22	13.59	18.20	13.72	13.00	3.59	11.97	-1.43	5.66	9.74	7.86	0.66	9.60	3.92	5.61	6.10	6.58
Lung	9.30	15.18	none	10.88	10.01	2.69	13.10	-2.22	2.64	10.28	6.80	-0.48	10.75	4.16	5.01	6.98	7.08
Lung	9.18	19.74	18.14	12.33	14.71	4.60	12.99	-2.21	2.53	10.95	7.98	0.57	10.94	3.69	6.28	6.68	6.06
Lung	8.80	13.08	18.53	11.29	11.28	2.57	13.01	-1.23	4.96	9.92	9.48	1.15	11.71	4.94	3.67	9.97	10.84
Lung	9.72	none	15.07	16.57	12.68	4.32	11.83	-3.49	2.34	9.49	8.98	0.29	10.50	4.40	4.66	8.43	8.90
Spleen	9.29	18.27	7.03	17.01	15.73	7.20	11.98	-0.85	-1.10	6.22	4.32	-2.08	6.75	3.16	2.58	4.19	3.29
Spleen	8.36	23.74	5.81	12.23	10.78	4.47	10.00	-0.92	-1.34	6.37	4.98	-2.86	7.43	2.90	4.87	5.84	3.87
Spleen	10.40	21.19	6.74	14.20	13.37	6.32	13.00	-0.60	-1.03	7.76	5.49	-2.85	7.91	3.20	2.11	4.96	3.95
Spleen	9.12	none	13.26	14.73	none	4.87	11.18	-0.29	-0.29	6.80	6.44	-2.00	7.70	3.41	-0.40	5.67	4.23
Spleen	10.19	15.33	15.62	13.19	12.12	5.49	11.15	-1.79	-0.63	7.70	6.21	-1.94	8.14	4.18	0.44	6.20	6.07
Tymus	8.13	24.10	10.03	13.46	none	4.62	13.83	0.08	0.72	6.84	6.35	-1.55	8.23	2.65	2.27	5.19	3.11
Tymus	7.50	16.11	9.80	10.78	11.38	1.52	11.16	0.32	0.94	8.35	7.21	-1.44	8.53	3.74	2.64	6.59	2.77
Tymus	8.41	18.50	14.47	11.73	11.48	2.69	12.90	1.08	1.68	9.93	6.69	-1.96	9.39	4.35	3.29	11.32	10.91
Tymus	9.77	13.43	11.57	8.89	8.53	-0.18	9.31	0.16	2.77	7.88	6.16	-3.68	7.72	3.47	-0.32	8.67	8.56
Tymus	7.14	13.92	11.52	11.39	10.65	3.31	11.04	-0.89	0.98	8.69	8.47	-1.07	9.58	3.54	2.36	6.60	3.37

H	IFN-α	IFN-β	IFN-γ	IL-1β	IL-2	IL-4	IL-6	MHC-I	MHC-II	BAFF	CCL-19	CCL-21	TLR-7	TLR-3	β-defensin	RIG-1	MDA5
Blood	7.76	19.30	19.26	22.51	17.25	8.48	19.67	-3.62	2.99	9.19	17.44	12.22	11.26	6.92	8.29	8.29	3.39
Blood	8.14	15.63	16.80	16.15	13.25	6.75	15.80	-2.62	2.14	7.43	14.28	10.29	9.84	5.22	7.19	6.05	2.28
Blood	7.30	16.38	19.15	15.73	13.55	8.78	19.80	-1.96	4.30	9.29	13.22	8.97	11.40	8.62	6.42	12.42	8.34
Blood	7.27	16.54	none	17.70	13.54	8.01	16.01	-2.97	3.31	9.47	15.35	9.88	11.27	7.54	8.53	11.52	7.89
Blood	5.20	14.64	18.18	15.13	12.45	6.89	15.19	-3.05	2.29	7.47	13.35	9.04	10.44	5.20	6.10	6.30	3.36
Fabricius	13.60	22.04	16.15	15.80	12.50	5.36	15.67	1.23	2.29	12.37	8.98	0.54	11.11	5.03	14.65	8.49	8.31
Fabricius	11.94	14.53	14.13	12.90	9.89	2.94	12.13	0.80	1.44	10.44	10.39	2.46	7.82	6.00	11.71	8.76	6.58
Fabricius	7.91	23.35	15.18	13.99	14.57	4.61	12.16	1.31	2.36	10.81	11.27	1.12	8.52	6.80	10.43	9.06	7.84
Fabricius	7.30	22.33	12.30	13.66	13.10	3.44	13.68	0.72	3.33	10.13	10.81	1.53	6.59	6.05	9.79	8.71	5.83
Fabricius	10.19	22.06	11.70	15.36	8.40	2.28	10.08	-1.36	2.03	9.62	6.00	-2.12	9.44	2.75	7.65	6.98	8.49
Hardrain	15.09	24.46	16.11	21.10	13.20	5.67	6.70	-0.30	3.80	11.90	8.25	2.01	12.03	3.15	12.94	4.08	4.41
Hardrain	14.47	13.93	none	12.38	10.55	2.47	6.12	2.22	4.79	11.64	8.96	1.44	10.59	4.72	7.46	5.56	8.35
Hardrain	14.45	22.78	15.66	18.51	17.45	5.50	5.64	1.86	5.00	10.01	10.28	2.68	11.11	3.35	9.92	4.63	6.33
Hardrain	14.04	22.51	16.07	18.69	13.26	6.76	6.92	2.12	6.08	11.54	10.66	5.59	12.41	4.58	10.88	4.73	6.19
Hardrain	7.27	none	13.38	9.78	13.82	-0.92	4.48	-0.04	6.91	11.01	8.75	-1.02	9.46	2.29	6.21	5.74	4.63
Kidney	12.00	15.76	14.72	21.87	12.17	4.27	15.32	1.38	4.94	14.03	11.85	2.36	15.14	5.11	13.85	9.83	7.30
Kidney	13.38	19.00	14.38	14.86	10.25	4.52	14.67	2.75	6.96	15.53	11.90	3.25	14.47	5.50	10.67	10.95	8.99
Kidney	9.89	18.36	22.23	21.16	15.43	6.22	18.18	3.32	7.07	14.75	12.62	4.48	15.88	7.72	10.37	11.22	9.56
Kidney	8.08	24.23	14.06	14.29	12.17	4.53	12.60	2.54	6.75	12.50	10.26	1.94	13.07	4.24	6.36	10.48	7.97
Kidney	9.93	21.65	none	13.53	12.52	3.82	16.67	3.19	8.49	13.88	11.95	2.92	14.01	5.84	8.26	14.13	13.41
Liver	9.00	23.38	11.00	13.28	12.89	4.28	13.45	1.74	4.36	10.69	9.26	0.33	11.33	5.97	9.61	8.34	6.98
Liver	13.16	12.97	9.50	20.66	10.13	1.62	12.11	1.37	3.66	14.11	10.72	0.48	11.28	5.41	6.30	9.77	6.86
Liver	10.55	26.04	12.09	15.30	20.41	6.01	19.02	4.21	6.03	12.12	13.32	3.18	13.45	8.09	8.62	9.87	7.90
Liver	8.26	15.70	12.16	16.36	12.29	4.29	15.23	2.43	4.66	10.84	10.25	1.06	11.89	5.96	7.87	8.61	6.77
Liver	9.43	24.66	9.49	13.46	13.86	4.02	12.50	1.84	4.51	13.45	10.57	0.47	13.44	6.69	8.03	9.10	6.63
Lung	8.87	17.28	11.87	10.56	none	3.54	9.81	-3.14	2.40	9.09	7.09	-0.47	10.69	4.76	6.64	8.89	8.32
Lung	13.11	22.70	13.47	18.20	12.16	3.43	10.85	-2.06	2.56	9.68	8.51	-0.38	10.45	3.64	5.79	8.20	8.68
Lung	9.94	18.05	14.85	17.23	14.32	4.89	11.77	-2.11	2.53	10.44	7.23	-0.26	10.29	4.99	3.80	10.79	11.34
Lung	10.96	14.21	13.36	18.29	23.09	4.00	10.32	-1.39	3.51	9.35	7.64	-0.36	9.53	3.87	6.65	8.48	10.66
Lung	7.88	16.20	20.74	13.53	12.52	3.12	12.17	-3.67	3.06	9.16	7.51	-0.52	9.81	3.65	5.34	6.78	6.27
Spleen	9.05	23.09	15.41	11.58	12.46	4.01	9.79	-2.43	-1.83	6.96	5.21	-3.07	7.12	2.17	6.59	6.23	5.63
Spleen	11.90	23.00	11.06	14.18	13.02	5.01	11.92	-0.56	-0.36	7.80	5.04	-2.15	7.23	2.92	0.75	6.37	5.15
Spleen	9.46	19.66	10.21	13.85	19.74	6.87	11.81	-0.51	-0.24	8.73	7.00	-0.89	7.83	4.02	0.35	6.38	5.54
Spleen	9.63	15.99	14.21	18.50	13.88	5.71	11.38	-1.42	-0.64	7.16	5.59	-1.90	7.14	2.51	2.04	6.18	5.75
Spleen	6.26	22.18	10.53	11.20	10.27	2.07	8.93	-2.87	-1.88	6.27	4.92	-3.85	5.45	1.53	2.51	4.77	3.07
Tymus	14.97	19.71	12.93	24.12	12.30	2.70	15.14	-1.00	0.45	9.25	6.50	-2.98	9.07	4.30	3.83	9.39	9.51
Tymus	9.73	19.65	13.46	11.02	9.25	-0.33	9.92	-0.60	1.92	8.60	7.25	-1.26	8.10	3.08	2.63	7.08	3.97
Tymus	7.28	17.82	12.22	16.50	16.84	3.26	12.13	-0.35	2.45	9.49	8.66	-0.40	8.79	4.58	3.86	8.70	4.97
Tymus	9.55	none	22.75	13.13	12.07	3.81	14.00	-0.17	2.24	9.12	7.42	-1.03	9.50	4.45	2.08	7.55	5.64
Tymus	8.93	19.25	none	12.37	10.70	2.97	12.91	-0.41	1.39	8.99	8.18	-0.92	8.57	3.93	4.07	9.38	9.25
